# Supplementary material for: The Drosophila RNA Helicase Belle (DDX3) Non-Autonomously Suppresses Germline Tumorigenesis Via Regulation of a Specific mRNA Set
Source: Cells. 2020 Feb 26;9(3):550. doi: 10.3390/cells9030550 (PMC7140462; doi:10.3390/cells9030550)
Supplement: Supplementary file 1 [file cells-09-00550-s001.zip › cells-679442-proof supplementary/Supplementary material.pdf]

Supplementary material for research article
The Drosophila RNA helicase Belle (DDX3) non-autonomously suppresses germline tumorigenesis via regulation of a specific mRNA set

Alexei A. Kotov[†1], Baira K. Godneeva[†1], Oxana M. Olenkina[1], Vladimir E. Adashev[1], Mikhail V. Trostnikov[1] and Ludmila V. Olenina[1]*

* Corresponding author: Ludmila V. Olenina,
olenina_ludmila@mail.ru

This file includes:

Figs. S1 to S10 and Suppl. Figure Legends

Legends for Tables S1-S4.

Figure S1

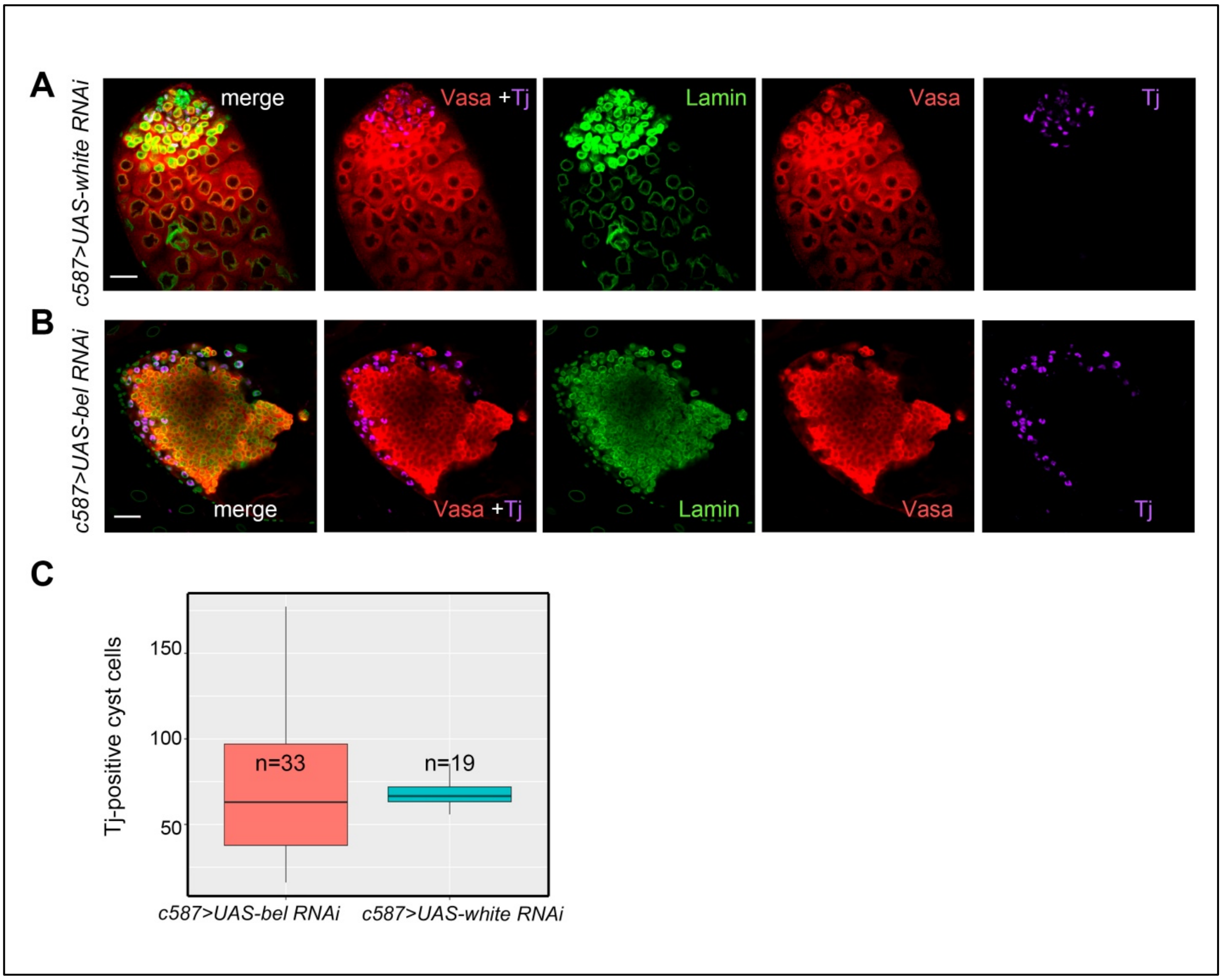

Figure S1. Comparative immunostaining analysis of the testes of males *c597-GAL4>UAS-bel RNAi* and *c587-GAL4>UAS-white RNAi*. Testes of newly eclosed control *c587-GAL4>UAS-white RNAi* (**A**) and males with *belKD* in cyst cells (**B**) were immunofluorescently stained with anti-Vasa (red), anti-Lamin (green), and anti-Tj (violet) antibodies. Internal confocal slices of the immunostained whole-mount fixed testis preparations are shown. Scale bars are 30 µm. (**A**) Testes of the control *c587-GAL4>UAS-white RNAi* line represented the wild-type phenotype. (**B**) More than one half of the analyzed *c587-GAL4>UAS-bel RNAi* testes (63.6%) contained tumor-like clusters of early germ cells with Tj-stained somatic cyst cells found to be segregated from germ cells. Another fraction of *c587-GAL4>UAS-bel RNAi* testes mainly represented a mosaic phenotype (30.0%) and contained cysts of spermatogonia and spermatocytes along with tumor-like clusters (not shown). (**C**) Tj-stained cyst cells insignificantly differ in number in the testes upon *belKD* in cyst cells compared to control ones. The boxplot diagram shows the number of Tj-positive early cyst cells in *c587-GAL4>UAS-bel RNAi* and control *c587-GAL4>UAS-white RNAi* testes (mean 77.85 ± 45.01 cells (n = 33) versus 69.84 ± 8.73 cells (n = 19) in the control; p-value = 0.775). Statistical analysis was performed by Wilcoxon/Mann–Whitney two-sided test. The bold lines on the boxes mean median values.

Figure S2

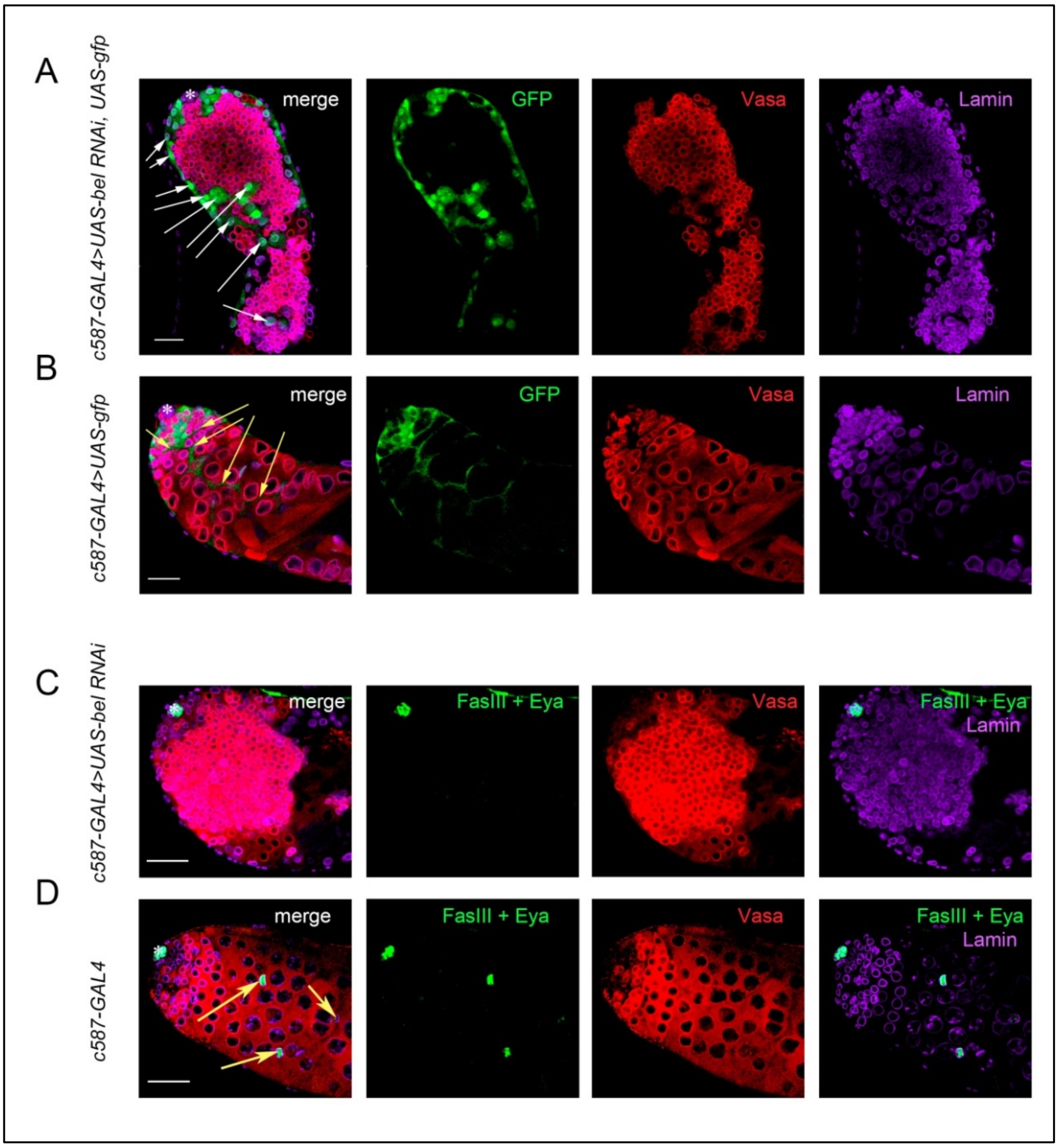

Figure S2. Peculiarities of cyst cell morphology of the testes with *belKD* in cyst cells. (**A-B)** Somatic cyst cells did not encapsulate early germ cells in the *c587-GAL4>UAS-bel RNAi, UAS-gfp* testes**.** Testes of young males (0-1 day after eclosion) were stained with anti-Vasa (red) and anti-Lamin (violet) antibodies. Cyst cells were marked by GFP (green). Internal confocal slices of the whole-mount fixed testis preparations are shown. The apical tips of the testes are oriented leftward. White asterisks indicate hub positions. Scale bars are 30 µm. (**A**) Testes of *c587-GAL4>UAS-bel RNAi, UAS-gfp* males contain cyst cells (white arrows) which are located separately from germ cell clusters (n=17). (**B**) Testes of control *c587-GAL4>UAS-gfp* males exhibited the wild-type phenotype with flat and elongated cyst cells (yellow arrows) enveloping germ cells in the cysts (n=24). (**C-D**) Strong reduction of Eya-marked mature cyst cells was observed in *c587-GAL4>UAS-bel RNAi* testes. Testes of young males (0-1 day after eclosion) were immunostained with anti-α-Eya (green), anti-Fas III (green), anti-Vasa (red), and anti-Lamin (violet) antibodies. White asterisks indicate hub positions. Scale bars are 40 µm. (**C)** Testes of *c587-GAL4>UAS-bel RNAi* males often did not contain Eya-marked cyst cells. (**D**) Testes of control *c587-GAL4* males exhibited the presence of Eya-stained mature cyst cells (yellow arrows) at a distance from the apical tip. For statistical data, see Figure 1G.

Figure S3

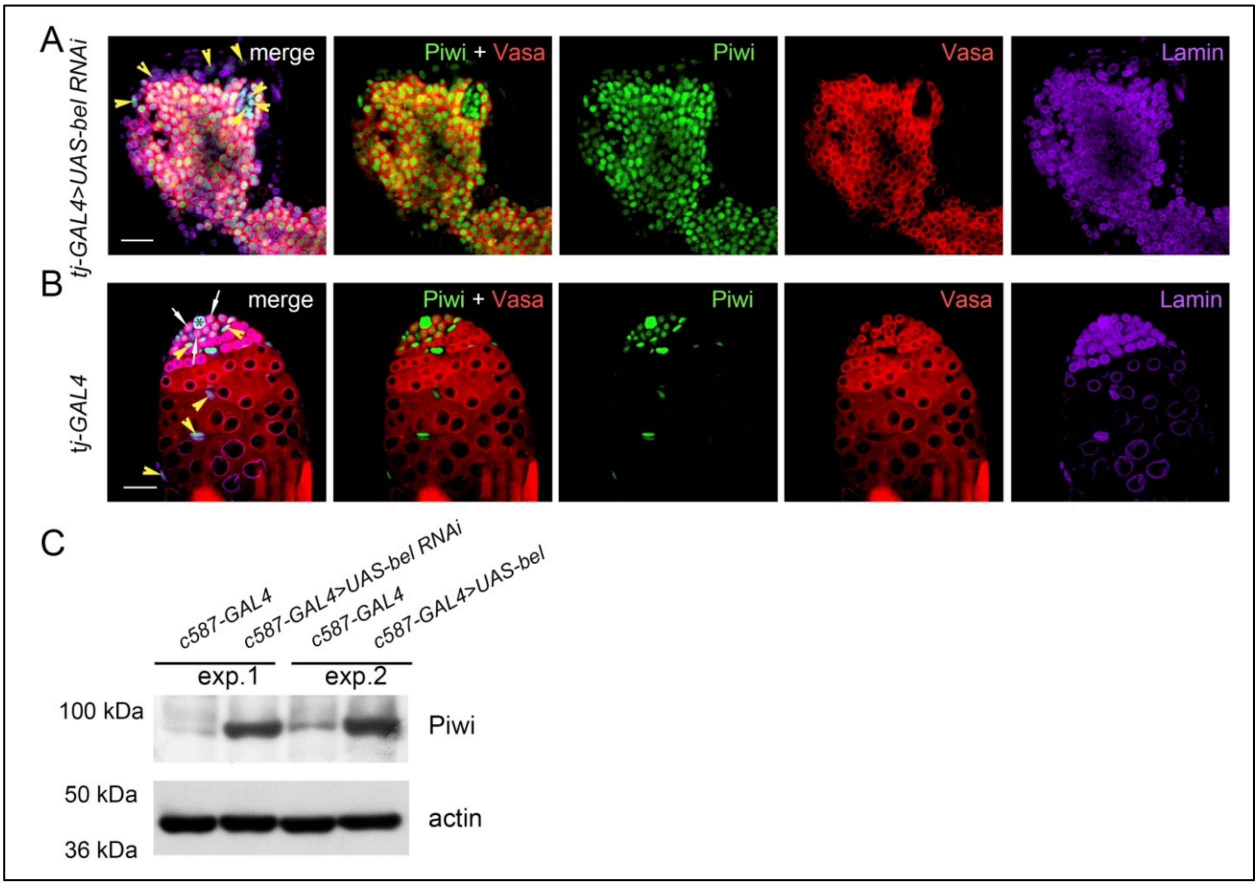

Figure S3. Piwi protein is overexpressed in the clusters of germ cells. (**A, B**) Testes of young males (0-1 day after eclosion) were stained with anti-Vasa (red), anti-Piwi (green), and anti-Lamin (violet) antibodies. Internal confocal slices of the whole-mount fixed testis preparations are shown. Scale bars are 30 µm. (**A**) Testes of *tj-GAL4>UAS-bel RNAi* males contain somatic cyst cells (yellow arrowheads) and the germ cell of the clusters all of which are Piwi-positive. (**B**) Testes of control *tj-GAL4* males contain Piwi-positive somatic cyst cells (yellow arrowheads) as well as GSCs (white arrows) and their immediate daughter cells, goniablasts. Grey asterisk indicates hub positions. (**C**) Western blot analysis of Piwi in testis lysates of *c587-GAL4>UAS-bel RNAi* and *c587-GAL4* flies. Loading was 40 µg per lane. Anti-actin antibodies were used as a loading control. The results of two independent experiments are shown.

Figure S4

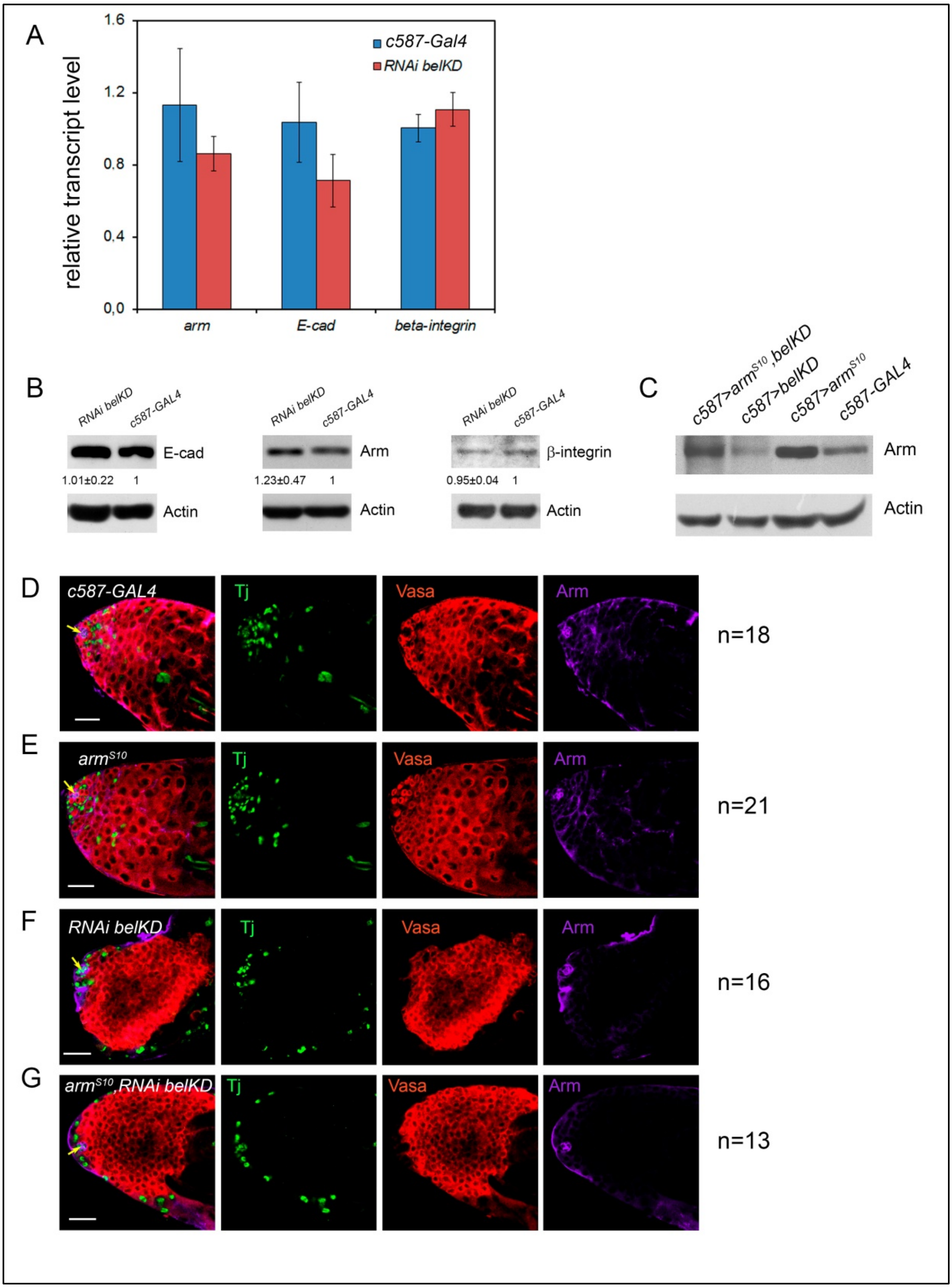

Figure S4. Analysis of expression of components of cell adhesion complexes. (**A**) RT-qPCR analysis of transcription of *E-cadherin*, *β-integrin*, *β-catenin* (*arm*) in the testes of *c587-GAL4>UAS-bel RNAi* and control *c587-GAL4* flies. Differences between the knockdown and control samples were found insignificant for all pairs ($p > 0.05$, Student's test). (**B**) Western blots of testis lysates probed with anti-E-cadherin, anti-β-integrin, and anti-Arm antibodies. Anti-actin antibodies were used as loading controls. Mean values and standard errors are presented for at least three independent experiments. The differences between the experimental and control

preparations are insignificant in all cases ($p > 0.05$, Student's t-test). (**C)** Western blot analysis of lysates of the testes with overexpression of the transgenic *arm*^{S10} copy and *belKD* in cyst cells and control line testes. (**D-G**) Overexpression of the transgenic *arm*^{S10} copy in cyst cells did not restore the wild-type testis phenotype. Testes of young males were stained with anti-Vasa (red), anti-Tj (green), and anti-Arm (violet) antibodies. Internal confocal slices of the whole-mount fixed testis preparations are shown. Yellow arrows indicate hub positions. Scale bars are 30 µm. (**D**) The testes of *c587-GAL4* flies were used as a wild-type control. (**E**) The testes of *c587-GAL4>UAS-arm*^{S10} flies did not exhibit defects of germ cell differentiation. (**F**) The testes of *c587-GAL4>UAS-bel RNAi* males contained clusters of undifferentiated early germ cells. (**G**) Ectopic expression of *arm*^{S10} copy in cyst cells in the background of *RNAi belKD* (*c587-GAL4>UAS-bel RNAi;UAS-armS10)* did not lead to the restoration of the wild-type testis phenotype.

Figure S5

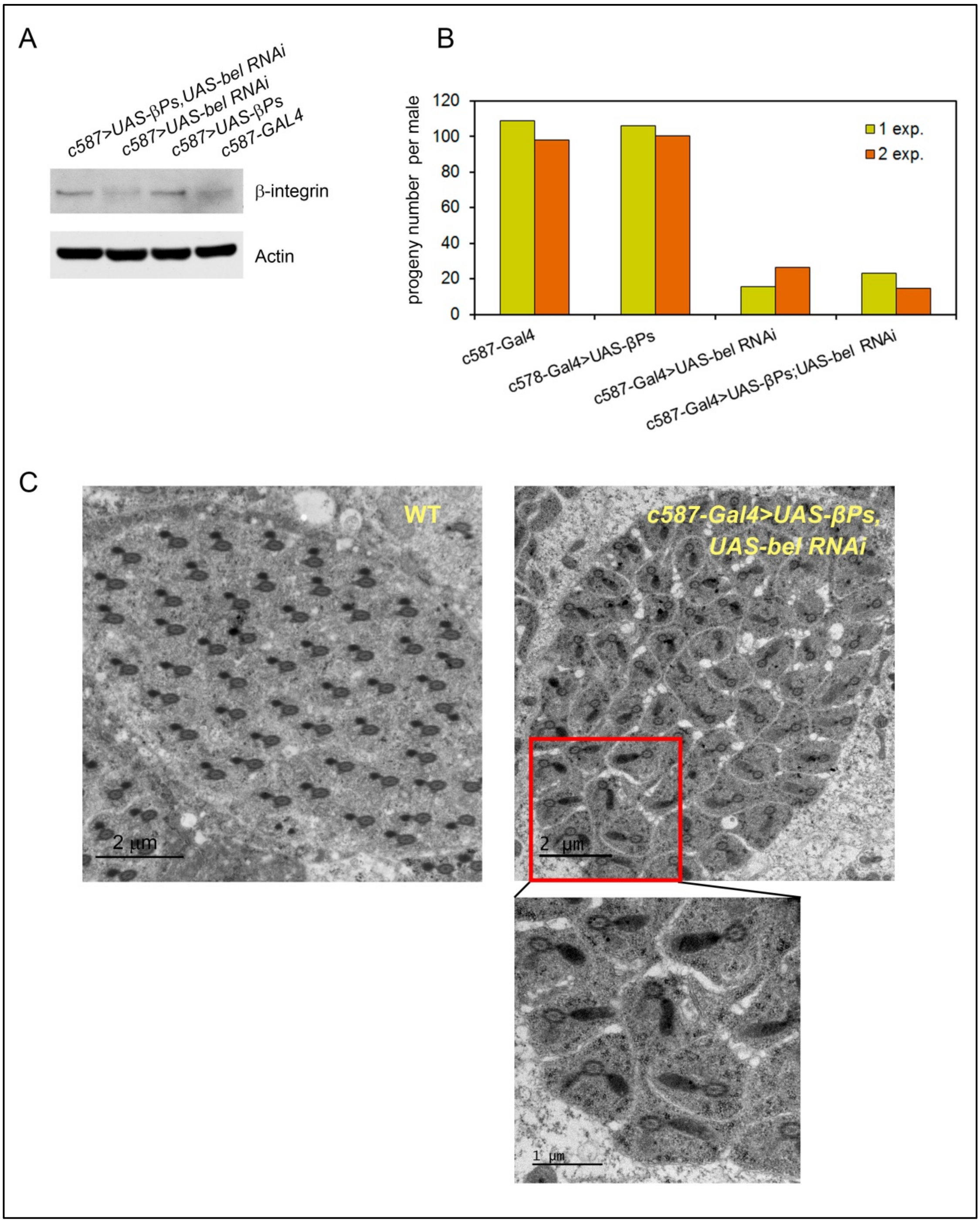

Figure S5. Analysis of testes with ectopic expression of *βPS (β-integrin)* copy in cyst cells in the background of *RNAi belKD.* (**A**) Western blot analysis of lysates of the testes with overexpression of the transgenic copy *β-integrin* and *belKD* in cyst cells and control lines. (**B**) Male fertility tests were performed as described in the Materials and Methods. Data from two independent experiments are presented. Ectopic expression of the additional *β-integrin* copy in cyst cells in the background of *RNAi belKD* (*c587-GAL4>UAS-βPS; UAS-bel RNAi)* did not lead to restoration of male fertility. (**C**) Transmission electron microscopy of the late stages of spermatogenesis. In contrast to normal spermiogenesis in the wild-type testes, the testes of *c587-GAL4>UAS-βPS; UAS-bel RNAi* males exhibited cyst integrity disorders and disorganized mitochondrial derivatives of spermatids in elongation; developing spermatids were randomly oriented.

Figure S6

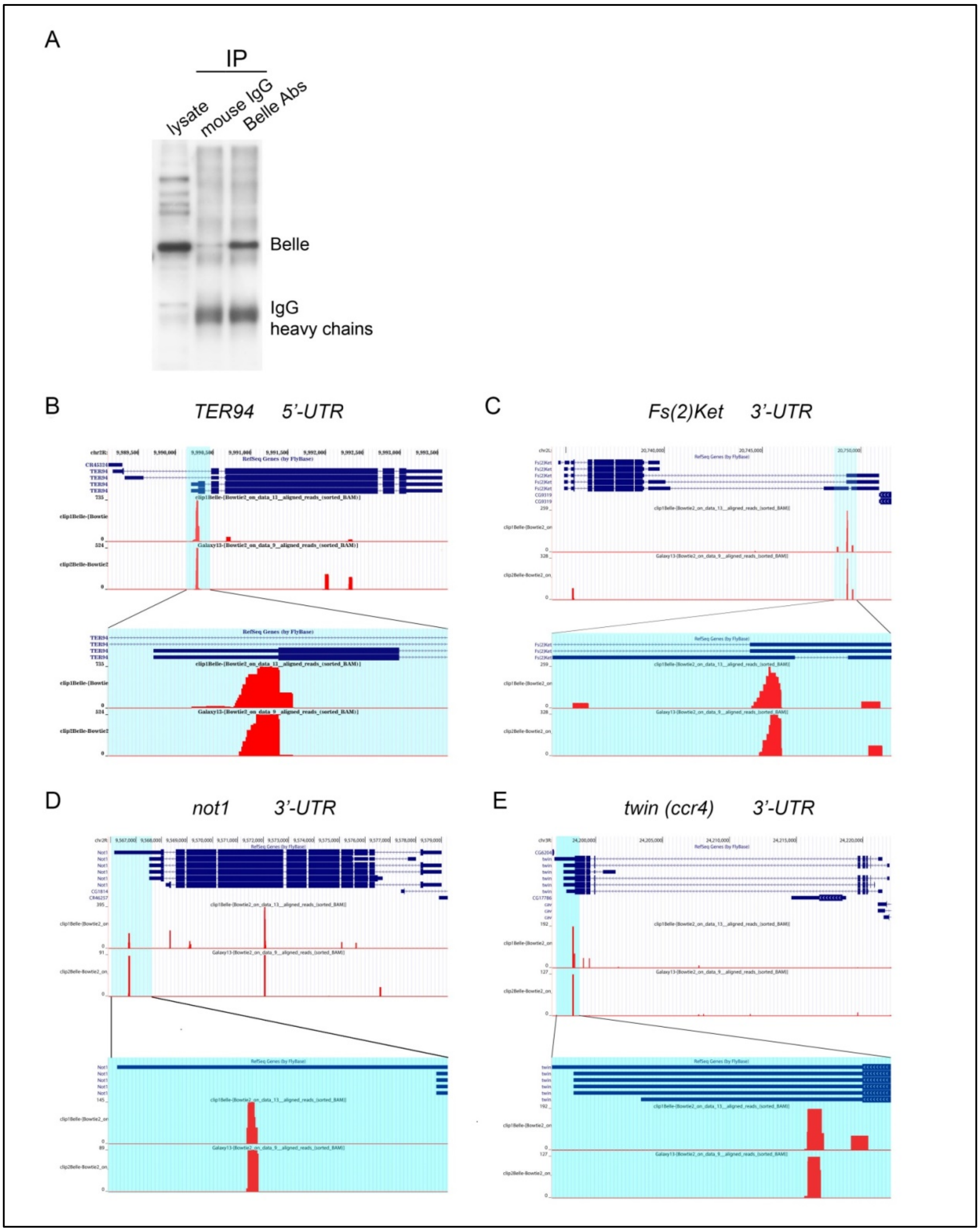

Figure S6. Preparation and analysis of Belle CLIP-seq libraries. (**A**) Western blot analysis of immunoprecipitation of Belle-RNA-complexes using anti-Belle antibodies. (**B-E**) Examples of genomic mapping of peaks from two Belle CLIP-seq libraries. Peaks from CLIP-seq libraries (red graphs) mapped to 5'-UTR or 3'-UTR of *not1*, *twin*, *Ter94*, and *Fs(2)ket* genes are presented in the *dm6* Genome Browser window. Fragments in cyan boxes are shown in the bottom as enlarged images. Reads were mapped to the *dm6* genome assembly using Bowtie 2 software after adapter removal and quality filtering.

Figure S7

A

	GO term	Gene number	Fold enrichment	p-value
Molecular functions	GO:0004176~ATP-dependent peptidase activity	2	19.86	0.096258
	GO:0004471~malate dehydrogenase (decarboxylating) (NAD+) activity	2	19.86	0.096258
	GO:0005089~Rho guanyl-nucleotide exchange factor activity	3	5.73	0.094863
	GO:0048102~autophagic cell death	4	3.68	0.093960
	GO:0000209~protein polyubiquitination	4	3.68	0.093960
	GO:0005884~actin filament	3	5.77	0.093747
	GO:0010906~regulation of glucose metabolic process	7	2.25	0.089864
	GO:0008157~protein phosphatase 1 binding	3	5.96	0.088693
	GO:0005811~lipid particle	10	1.88	0.084985
	GO:0051646~mitochondrion localization	2	22.79	0.084323
	GO:0002121~inter-male aggressive behavior	4	3.86	0.083808
	GO:0030529~intracellular ribonucleoprotein complex	3	6.23	0.082112
	GO:0016887~ATPase activity	7	2.32	0.080748
	GO:0006096~glycolytic process	3	6.33	0.080084
	GO:0051998~protein carboxyl O-methyltransferase activity	2	24.82	0.077774
	GO:0030707~ovarian follicle cell development	5	3.10	0.077175
Cellular component	GO:0030150~protein import into mitochondrial matrix	3	6.57	0.074986
	GO:0007601~visual perception	4	4.07	0.074162
	GO:0003779~actin binding	6	2.66	0.074134
	GO:0006814~sodium ion transport	4	4.14	0.071064
	GO:0005912~adherens junction	4	4.16	0.070405
	GO:0045010~actin nucleation	2	28.49	0.068045
	GO:0040023~establishment of nucleus localization	2	28.49	0.068045
	GO:0055085~transmembrane transport	10	1.96	0.067980
	GO:0005819~spindle	4	4.24	0.067088
	GO:0005622~intracellular	11	1.88	0.067025
	GO:0007015~actin filament organization	5	3.39	0.059181
	GO:0016322~neuron remodeling	4	4.75	0.051112
	GO:0007005~mitochondrion organization	4	4.95	0.045993
	GO:0006402~mRNA catabolic process	3	9.00	0.042689
	GO:0007189~adenylate cyclase-activating G-protein coupled receptor signaling pathway	3	9.00	0.042689
Biological process	GO:0045727~positive regulation of translation	3	9.50	0.038630
	GO:0006101~citrate metabolic process	2	56.97	0.034618
	GO:0035556~intracellular signal transduction	6	3.38	0.031776
	GO:0005741~mitochondrial outer membrane	4	5.77	0.031074
	GO:0006839~mitochondrial transport	3	10.68	0.030994
	GO:0005875~microtubule associated complex	12	2.08	0.029412
	GO:0005856~cytoskeleton	5	4.26	0.029163
	GO:0008587~imaginal disc-derived wing margin morphogenesis	4	6.51	0.022770
	GO:0051082~unfolded protein binding	5	4.68	0.021300
	GO:0035023~regulation of Rho protein signal transduction	3	13.15	0.020846
	GO:0005739~mitochondrion	17	1.85	0.020729
	GO:0007409~axonogenesis	5	4.75	0.020553
	GO:0042787~protein ubiquitination involved in ubiquitin-dependent protein catabolic process	6	4.07	0.015656
	GO:0016319~mushroom body development	6	4.07	0.015656
	GO:0016567~protein ubiquitination	8	3.12	0.014060
	GO:0007291~sperm individualization	5	5.48	0.012700
	GO:0042026~protein refolding	3	17.09	0.012447
	GO:0015450~P-P-bond-hydrolysis-driven protein transmembrane transporter activity	4	8.63	0.010433
	GO:0004842~ubiquitin-protein transferase activity	10	2.77	0.009874
	GO:0031307~integral component of mitochondrial outer membrane	3	19.48	0.009468
	GO:0005742~mitochondrial outer membrane translocase complex	3	19.48	0.009468
	GO:0007283~spermatogenesis	9	3.17	0.007506
	GO:0005759~mitochondrial matrix	7	4.60	0.003982
	GO:0015141~succinate transmembrane transporter activity	3	29.79	0.003837
	GO:0015137~citrate transmembrane transporter activity	3	37.24	0.002333
	GO:0005737~cytoplasm	46	1.54	0.002321
	GO:0005524~ATP binding	28	1.85	0.002031
	GO:0071422~succinate transmembrane transport	3	42.73	0.001778
	GO:0004177~aminopeptidase activity	6	10.27	0.000246
	GO:0006458~'de novo' protein folding	4	32.56	0.000174
	GO:0030145~manganese ion binding	6	17.52	0.000016
	GO:0006626~protein targeting to mitochondrion	7	13.75	0.000009
	GO:0008235~metalloexopeptidase activity	6	21.28	0.000005

0 50

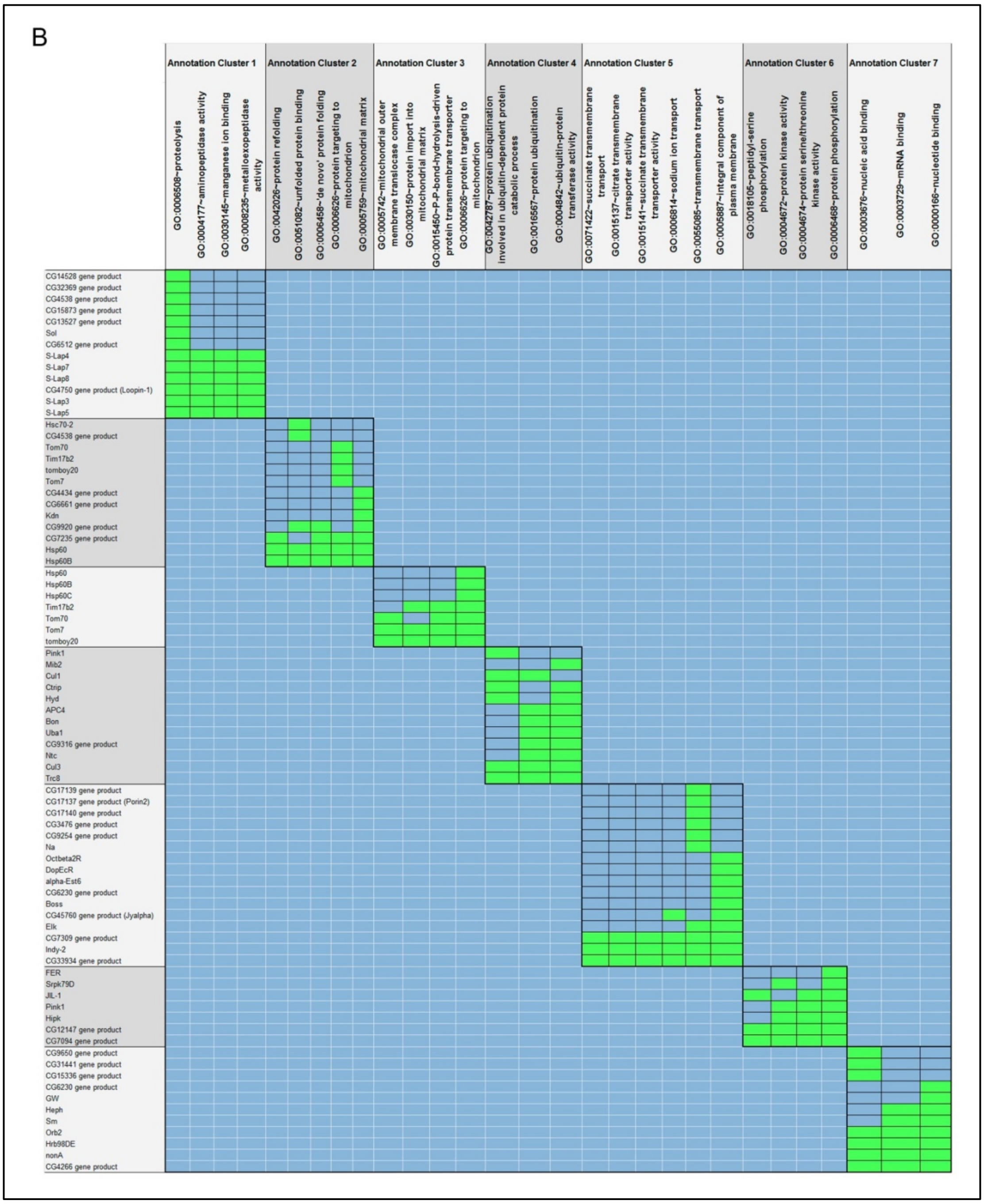

Figure S7. Gene ontology analysis of Belle mRNA targets in the testes using DAVID Bioinformatics Resource v6.8. (**A**) Gene number, fold enrichment, and p-value are shown for GO categories. Fold enrichment indicates the magnitude of enrichment for genes from our list included in a certain GO category vs. enrichment of the genes of this category in the *D. melanogaster* genome. Enrichment values more than 1.5 for subsets of 5 and more genes are marked by red. Only 64 genes were annotated by DAVID, the rest of the genes from Belle target list were not included in the output. Smaller p-values mean more significant results; the default cutoff for p-value was 0.1. (**B**) Map of GO functional annotation clustering of Belle mRNA targets. Green color bars indicate proteins included in a GO-category for cluster membership, blue color bars indicate proteins that are not included. Only targets included in at least one cluster are presented here.

Figure S8

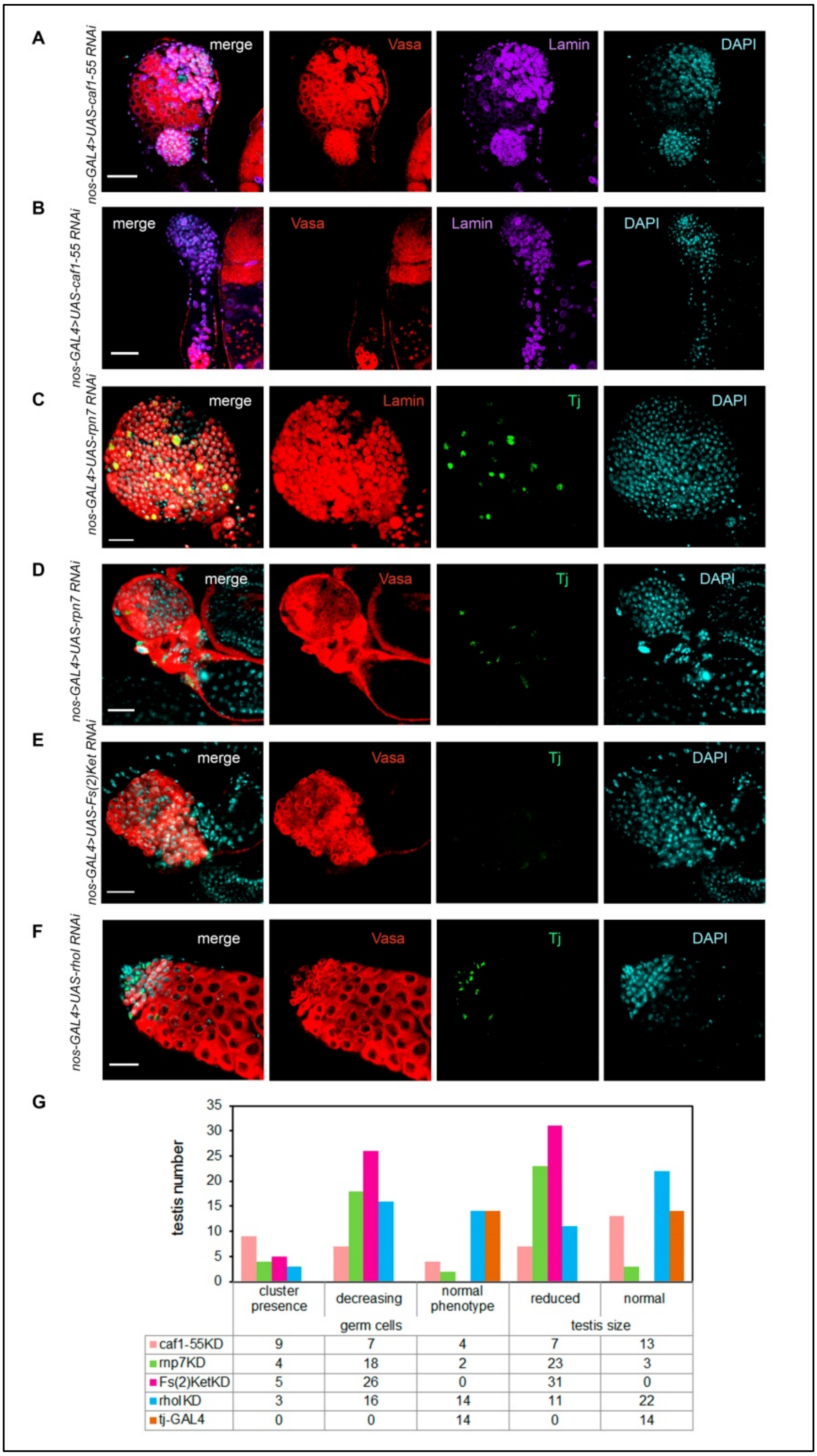

Figure S8. Immunostaining analysis of the testes of flies with *RNAi* knockdowns of Belle target gene expression in somatic cyst cells using the *tj-GAL4* driver. (**A-F**) Testes of freshly eclosed males (0-2 days) were stained with anti-Vasa (red), anti-Lamin (violet), and anti-Tj (green) antibodies, and anti-Lamin (red (C)). Chromatin was stained by DAPI (blue) for indication of nuclei. Scale bars are 30 μm. (**A,B**) 45% of the *caf1-*

55KD testes (n=20) contain germ cell clusters. (**C,D**) 15% of the *rpn7KD* testes (n=26) also demonstrate germ cell cluster formation. (**E**) 16% of the *Fs(2)KetKD* testes (n=31) contain germ cell clusters. (**F**) 9% of the *rhoIKD* testes exhibit germ cell clusters (not shown), whereas 42% of the *rhoIKD* testes have the wild-type phenotype (n=33). (**G**) Summary graph of phenotypes exhibited by immunostained testes with *RNAi* knockdowns of selected genes in somatic cyst cells. The testes of *tj-GAL4* males (n=14) are used as control ones. All they possess the wild-type phenotype (not shown).

Figure S9

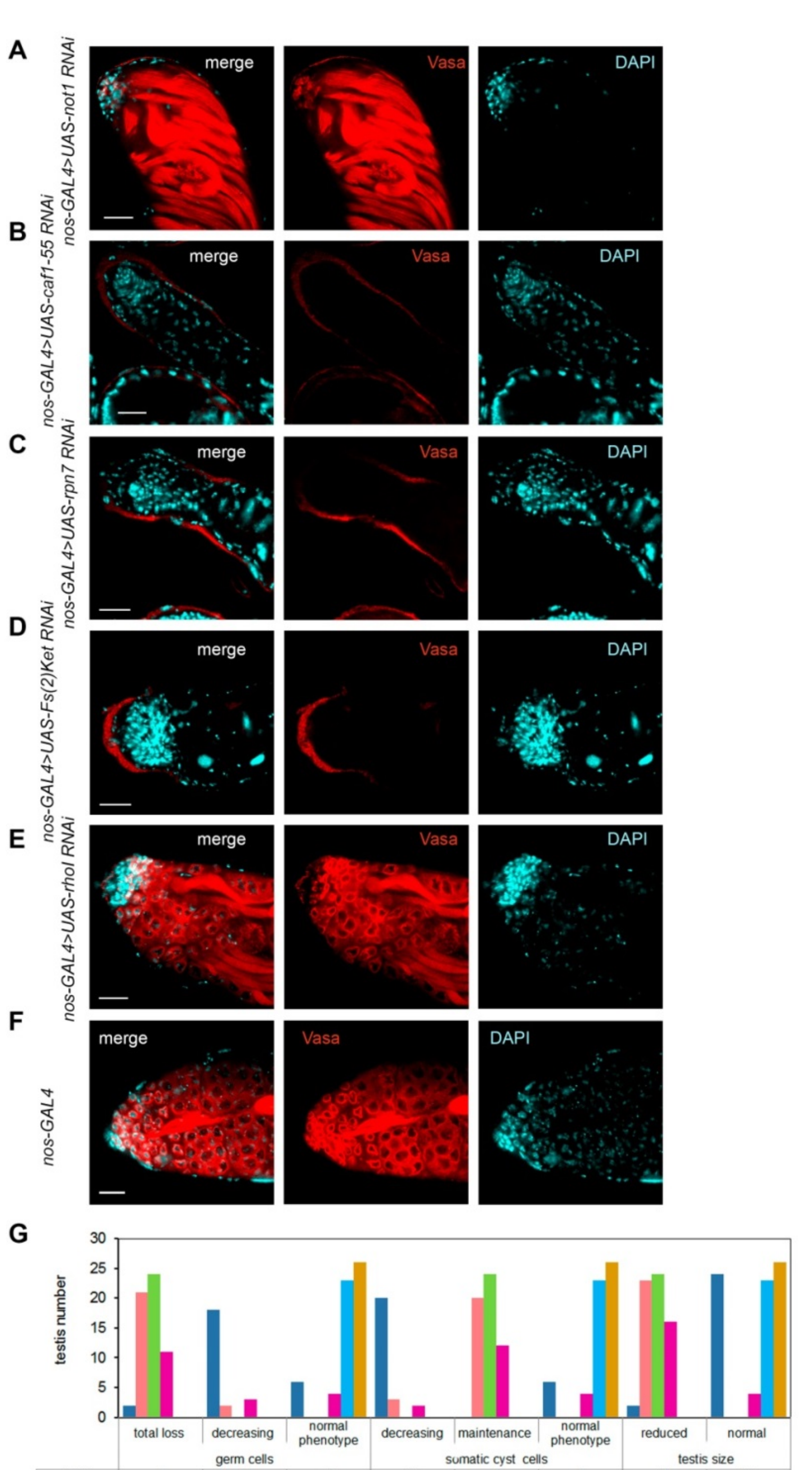

	germ cells			somatic cyst cells			testis size	
	total loss	decreasing	normal phenotype	decreasing	maintenance	normal phenotype	reduced	normal
not1KD	2	18	6	20	0	6	2	24
caf1-55KD	21	2	0	3	20	0	23	0
rnp7KD	24	0	0	0	24	0	24	0
Fs(2)KetKD	11	3	4	2	12	4	16	4
rholKD	0	0	23	0	0	23	0	23
nos-GAL4	0	0	26	0	0	26	0	26

Figure S9. Immunostaining analysis of the testes of flies with *RNAi* knockdowns of Belle target gene expression in the germline using the *nos-GAL4* driver. (**A-F**) Testes of freshly eclosed males (0-2 days) were stained with anti-Vasa (red) antibodies. Chromatin was stained by DAPI (blue) for indication of nuclei. Scale bars are 30 µm. (**A**) *not1KD* testes (n=26) exhibit significant germ cell and somatic cell decrease. (**B**) *caf1-*

55KD testes (n=23) exhibit a total germ cell loss, but maintenance of somatic testes cells. (**C**) *rpn7KD* testes (n=24) also demonstrate total germ cell loss and maintenance of somatic testes cells. (**D**) *Fs(2)KetKD* testes (n=20) possess somatic cells but generally lose germ cells. (**E**) *rhoIKD* testes (n=23) exhibit closely to wild-type phenotype. (**F**) The testes of *nos-GAL4* driver line (n=26) are used as control ones. All they possess a wild-type phenotype. (**G**) Summary graph of phenotypes exhibited by immunostained testes with *RNAi* knockdowns of selected genes in the germline and *nos-GAL4* driver male testes as control.

Figure S10

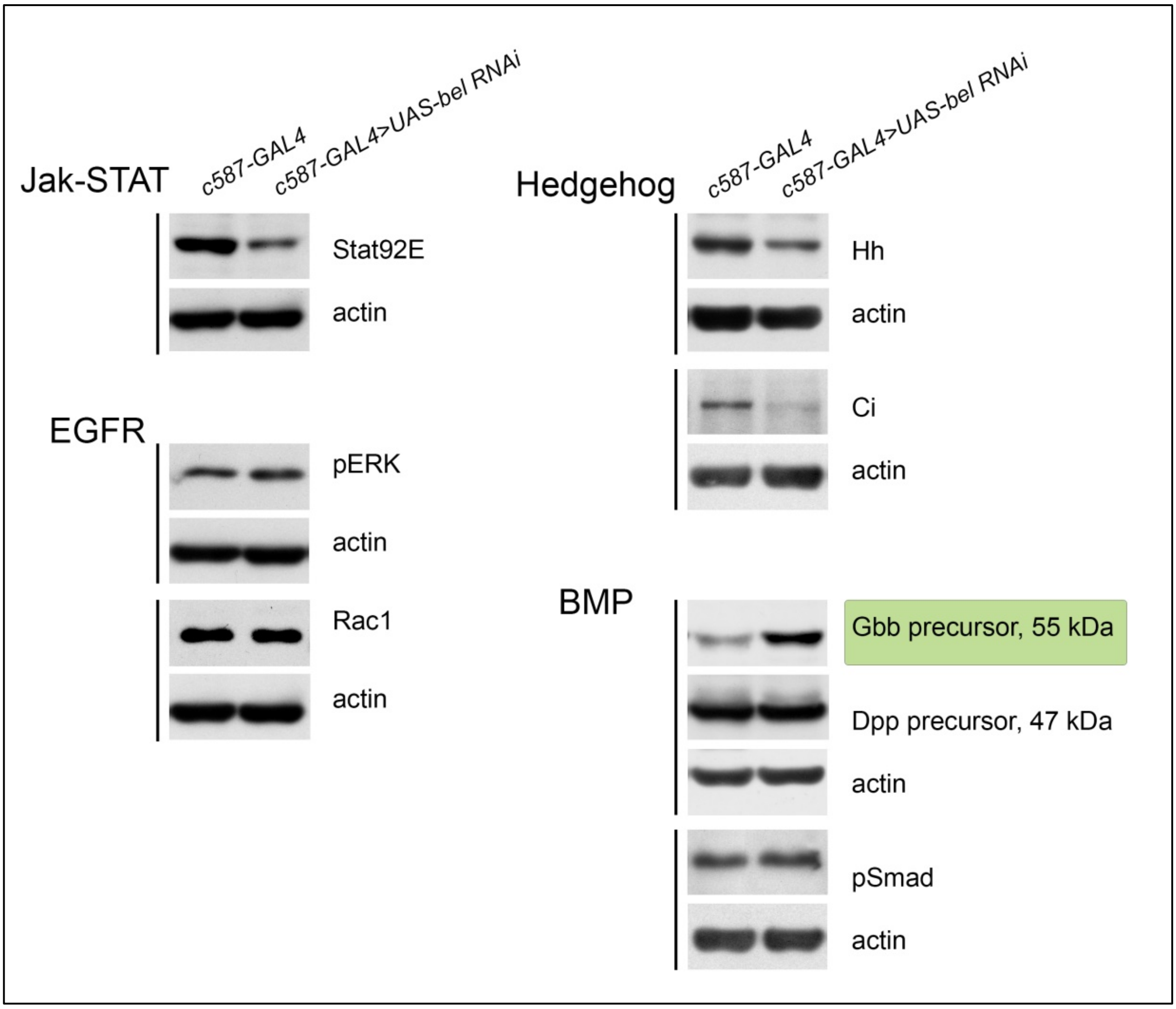

Figure S10. Western blot analysis of testis lysates with antibodies to key components of different signaling pathways. Analysis of testis lysates of *c587-GAL4>UAS-bel RNAi* flies and the control *c587-GAL4* line was performed. Vertical bars on the left indicate sets of blots with a common loading control (actin) shown at the bottom. All experiments were performed at least in triplicate. Gbb is highlighted in green boxing as a direct Belle target.

Supplementary Tables Legends

Table S1. List of Belle RNA targets determined by Piranha software with selection conditions p>0.87 for both CLIP1 and CLIP2.

Table S2. GO analysis of Belle RNA targets using DAVID Bioinformatics Resource v6.8.

Table S3. List of genes, knockdowns of which provide the morphological defects in the testes according to data of three independent screens and which determined in our CLIP analysis as putative mRNA targets of Belle. *RNAi* knockdowns of 17 genes determined in CLIP analysis lead to similar phenotypes in case of *belKD* in somatic cyst cells or in germ cells according to the results of at least a one screen (matched by yellow and orange color). Four genes, *not1, rpn7, rho1,* and *caf1-55*, knockdowns of which provide the same morphological defects in the testes according to data of three independent screenings are marked by orange color.

Table S4. STRING-based interaction clusters.
