# Supplementary material for: The Drosophila RNA Helicase Belle (DDX3) Non-Autonomously Suppresses Germline Tumorigenesis Via Regulation of a Specific mRNA Set
Source: Cells. 2020 Feb 26;9(3):550. doi: 10.3390/cells9030550 (PMC7140462; doi:10.3390/cells9030550)
Supplement: Supplementary file 1 [file cells-09-00550-s001.zip › cells-679442-proof supplementary/Table S1.pdf]

5UTR	3UTR	coding	ncRNA	intron
Arf51F	CG5432	14-3-3epsilon	bft	Arf79F
cep290	Act5C	Acp26Aa	CR43314	arm
Fuca	ACXA	Acp76A	CR30121	axo
CG12362	ACXC	AIF	CR31386	B4
CG1288	bon	AIF	CR31386	B4
CG1394	CG10055	alpha-Est6	CR42862	bon
CG14995	CG11125	APC4	CR43260	bru3
CG15219	CG11141	apolpp	CR43334	bs
CG15418	CG11779	apolpp	ban	byn
CG17140	CG12362	Arf102F	CR43334	c11.1
CG42284	CG13700	ATPCL	ban	CAH2
CG31178	CG13954	ave	CR43615	caps
CG33934	CG13739	CG9263	CR43627	CG10384
CG34312	CG13989	beta'COP	CR43651	CG10936
CG42558	CG14234	betaTub85D	mir-14	CG11873
CG4266	CG15124	betaTub85D	CR43761	CG12983
CG17344	CG15336	bor	CR43839	CG14372
CG44435	CG15337	boss	CR43906	CG14535
CG44435	CG15618	CaBP1	CR44031	CG15506
CG9133	CG1662	Caf1-55	CR44062	CG15630
CG9133	CG16782	Capr	CR44066	CG17684
Cul3	CG17097	Cchl	CR44267	CG17985
DopEcR	CG17139	Cct1	CR44833	CG2082
FER	CG17377	CG10252	CR45267	CG3168
for	CG1902	CG10407	CR45361	CG31709
larp	CG2127	CG10694	mir-1	CG32264
Menl-2	CG30271	rhi	CR45942	CG32719
MME1	CG31226	CG1138	mir-12	CG32809
Msp300	CG3124	CG11714	mir-304	CG33111
Octbeta2R	CG31441	CG12147	mir-9b	CG33111
Ppi1	CG31740	CG12177	iab-8	CG33158
Ppi1	CG32192	CG12395	mir-92a	CG34357
robl62A	CG32388	CG12547	mir-184	CG34357
S-Lap8	CG32572	CG12581	CR44206	CG3655
SCCRO3	CG32591	CG12861	mir-305	CG40470
sif	CG32651	CG12923	mir-318	CG40470
CG43201	CG34235	CG12983	mir-994	CG41378
CG43178	CG34315	CG13083	snoRNA:Psi18S-[illegible]	CG42321
CG43171	CG42847	CG13527	CR43960	CG42321
sol	CG42851	CG13611		CG42342
TER94	CG44434	CG13700		CG42346
toc	CG45154	CG13865		CG42389
tomboy20	CG4538	CG13978		CG42458
Uba1	CG4612	CG14013		CG42524
	CG4836	CG14043		CG42750
	CG5162	CG14204		CG43219
	CG6091	CG14215		CG43366
	CG6512	CG14354		CG43373
	CG7309	CG14355		CG43427
	CG7369	CG14355		CG43759
	CG7414	CG14480		CG44422
	CG9570	CG14528		CG45782
	Cpr	CG14537		CG5326
	Dbp80	CG14572		CG7544
	djl	CG14718		CG7990
	Dp1	CG14838		CG8399

eIF3-S8	CG14861	ckd
Eno	CG14861	Cngl
Fit1	CG14995	cpo
Fs(2)Ket	CG15109	cpx
glob1	CG15111	Ctr1A
Hipk	CG15144	cysu
kdn	CG15543	DIP-beta
l(1)G0320	CG15873	disco-r
Menl-1	CG15892	dnc
Mical	CG1640	DopEcR
nonA	CG16782	dpr11
Not1	CG16833	dpr13
omd	CG16972	dpr8
Past1	CG17139	dpy
psq	CG17140	ds
Rb97D	CG17217	ds
Rho1	CG17375	ds
CG43172	CG17470	Dscam2
CG43200	CG17625	EcR
CG46305	CG17652	ed
sm	CG17691	esn
sm	Rpt1	Frq2
Stim	CG18048	fz2
stops	CG18662	GEFmeso
sut4	CG1902	geko
Taf12L	CG1958	Gmap
twin	CG1988	Gmap
Yp1	CG1999	grp
	CG2127	gsb-n
	CG2137	hbn
	CG2921	hdc
	CG30196	heph
	CG30271	heph
	CG31010	hppy
	CG31294	Hr4
	CG31528	Indy
	CG31635	jigr1
	CG3164	Jupiter
	CG31751	Jupiter
	CG31784	Kat60
	CG31817	kek2
	CG31817	kl-3
	CG31819	kl-3
	CG31909	kl-3
	CG31948	ko
	CG32081	l(3)neo38
	CG32087	Lim1
	CG32117	Lim1
	CG32119	LRP1
	CG32119	LRR
	CG32181	mam
	ntc	Mical
	CG32369	mim
	CG43439	Myo81F
	CG32436	nord
	CG32459	Npl4
	CG32944	nuf
	eIF4G2	nuf
	eIF4G2	oc

CG33287	ocn
CG33290	CG43783
CG33490	Parp
Indy-2	PCB
CG30058	Pde1c
Orc3	pdm3
CG34425	Pli
CG3635	Ppn
CG4161	Ppr-Y
CG4198	Proc
CG42266	Prosap
CG30060	ptc
CG4631	Ptpmeg
CG42393	pyd
CG42557	Pzl
CG42684	RapGAP1
CG31788	Rbfox1
CG42788	Rbfox1
CG42827	Rbfox1
CG43121	rg
CG4329	CG15465
CG43319	RhoGAP1A
CG43343	RhoGEF3
CG43731	robo3
CG43755	RpS20
CG43755	Sb
CG44004	sdt
CG4434	Sema1a
CG4434	Sh
CG4434	shakB
CG44434	shep
CG44435	CG46320
CG44434	sima
CG45066	slo
CG4572	SLO2
CG46059	SLO2
CG4810	sm
CG4836	Smg5
CG4836	Snmp1
CG4836	spz3
CG4836	step
CG5122	Sulf1
CG5122	Ten-a
CG5144	timeout
CG5521	toc
CG5614	trv
CG6230	TyrRII
CG6304	Wdr62
CG6380	Xrp1
CG6404	
CG6412	
CG6420	
CG6441	
CG6441	
CG6481	
CG6652	
CG6661	
CG7059	
CG7069	

CG7094
CG7311
CG7408
CG7848
CG8042
CG8188
CG8520
CG8568
CG8746
CG9130
CG9130
CG9240
CG9253
CG9254
CG9316
CG9391
CG9393
CG9510
CG9590
CG9641
CG9650
CG9920
Chc
Ciao1
cnc
Col4a1
Col4a1
COX4
Cpsf160
ctrip
Cul1
Cyp12c1
Cyp4p3
Dbp73D
Dgk
dia
didum
disp
djl
Dnah3
DppIII
Drsl1
Ebp
ed
Ef1alpha48D
eIF-2beta
eIF-5A
eIF4E-5
Eip75B
Elk
EMC3
Erk7
Ext2
FBXO11
Frl
fry
fry
FucTB
garz

gbb
Gel
GluProRS
Gnf1
gw
HBS1
heph
His4r
hmw
Hrb98DE
Hsc70-2
Hsc70-3
Hsc70-5
Hsp60A
Hsp60B
Hsp60C
Hsp60C
hyd
hyd
Idgf4
Incenp
isopeptidase-T-3
JIL-1
JYalpha
KdelR
kl-2
Klp67A
lgs
loopin-1
loopin-1
mad2
mfas
mib2
CG31407
mino
Miro
Msp300
Mst77F
mud
na
Nop17l
Not1
Nph
CG32141
CG7768
Obp99d
ocn
Or67d
orb2
pbl
Pdi
CG8701
Pex11
Pink1
Pka-C3
Pmm45A
Pmm45A
Pms2
Porin2

Prat
S-Lap4
S-Lap4
S-Lap4
Rpn13R
RhoGEF3
RpL36
Rpn6
Rpn7
S-Lap2
S-Lap3
S-Lap3
S-Lap5
S-Lap7
S-Lap8
Sac1
Sac1
scpr-C
Sec31
I-3
shot
sick
sip2
SKIP
sle
Slmap
sns
Socs44A
Spase12
Srpk79D
ssh
ssh
Su(z)12
Taf1
CG2750
Tep2
tHMG1
Tim17b2
toc
Tom7
Tom70
Trc8
Trf4-2
TrpRS-m
Uba1
Ude
unc-5
upSET
Usp8
Vps8
Xrp1
Ziz
zormin

Supplementary Table S1. List of RNA targets determined by Piranha software with selection conditions p>0.87 for CLIP1 and with at least two unique reads in CLIP2
