# Supplementary material for: The Drosophila RNA Helicase Belle (DDX3) Non-Autonomously Suppresses Germline Tumorigenesis Via Regulation of a Specific mRNA Set
Source: Cells. 2020 Feb 26;9(3):550. doi: 10.3390/cells9030550 (PMC7140462; doi:10.3390/cells9030550)
Supplement: Supplementary file 1 [file cells-09-00550-s001.zip › cells-679442-proof supplementary/Table S2.pdf]

Functional Annotation Table DAVID v6.8

ID	Gene Name
FBgn0013749	ADP ribosylation factor at 102F(Arf102F)
FBgn0020236	ATP citrate lyase(ATPCL)
FBgn0002855	Accessory gland protein 26Aa(Acp26Aa)
FBgn0052707	Anaphase Promoting Complex subunit 4(APC4)
FBgn0031392	Apoptosis inducing factor(AIF)
FBgn0039104	CG10252 gene product from transcript CG10252-RA(CG10252)
FBgn0039147	CG10694 gene product from transcript CG10694-RA(CG10694)
FBgn0033397	CG10843 gene product from transcript CG10843-RA(Cyp4p3)
FBgn0037325	CG12147 gene product from transcript CG12147-RA(CG12147)
FBgn0030510	CG12177 gene product from transcript CG12177-RA(CG12177)
FBgn0250845	CG1288 gene product from transcript CG1288-RA(CG1288)
FBgn0033784	CG13322 gene product from transcript CG13322-RA(CG13322)
FBgn0034776	CG13527 gene product from transcript CG13527-RB(CG13527)
FBgn0033403	CG13739 gene product from transcript CG13739-RA(CG13739)
FBgn0035168	CG13889 gene product from transcript CG13889-RB(cep290)
FBgn0033405	CG13954 gene product from transcript CG13954-RC(CG13954)
FBgn0039518	CG13978 gene product from transcript CG13978-RA(CG13978)
FBgn0031659	CG14043 gene product from transcript CG14043-RA(CG14043)
FBgn0031032	CG14204 gene product from transcript CG14204-RB(CG14204)
FBgn0031052	CG14215 gene product from transcript CG14215-RA(CG14215)
FBgn0031065	CG14234 gene product from transcript CG14234-RA(CG14234)
FBgn0039611	CG14528 gene product from transcript CG14528-RA(CG14528)
FBgn0031954	CG14537 gene product from transcript CG14537-RA(CG14537)
FBgn0037828	CG14690 gene product from transcript CG14690-RA(tomboy20)
FBgn0038280	CG14861 gene product from transcript CG14861-RA(CG14861)
FBgn0035497	CG14995 gene product from transcript CG14995-RA(CG14995)
FBgn0034419	CG15111 gene product from transcript CG15111-RB(CG15111)
FBgn0030009	CG15336 gene product from transcript CG15336-RD(CG15336)
FBgn0031554	CG15418 gene product from transcript CG15418-RA(CG15418)
FBgn0039799	CG15543 gene product from transcript CG15543-RB(CG15543)
FBgn0031077	CG15618 gene product from transcript CG15618-RB(CG15618)
FBgn0035003	CG15873 gene product from transcript CG15873-RA(CG15873)
FBgn0030478	CG1640 gene product from transcript CG1640-RB(CG1640)
FBgn0030481	CG1662 gene product from transcript CG1662-RA(CG1662)
FBgn0029659	CG16782 gene product from transcript CG16782-RA(CG16782)
FBgn0265264	CG17097 gene product from transcript CG17097-RB(CG17097)
FBgn0069354	CG17137 gene product from transcript CG17137-RB(Porin2)
FBgn0260454	CG17139 gene product from transcript CG17139-RA(CG17139)
FBgn0260453	CG17140 gene product from transcript CG17140-RA(CG17140)
FBgn0040510	CG17176 gene product from transcript CG17176-RG(ACXA)
FBgn0032869	CG17470 gene product from transcript CG17470-RA(CG17470)
FBgn0035640	CG17498 gene product from transcript CG17498-RA(mad2)
FBgn0031361	CG17652 gene product from transcript CG17652-RB(CG17652)

FBgn0039993	CG17691 gene product from transcript CG17691-RC(CG17691)
FBgn0037435	CG18048 gene product from transcript CG18048-RA(CG18048)
FBgn0042712	CG1898 gene product from transcript CG1898-RB(HBS1)
FBgn0034689	CG2921 gene product from transcript CG2921-RA(CG2921)
FBgn0050058	CG30058 gene product from transcript CG30058-RA(CG30058)
FBgn0011725	CG31137 gene product from transcript CG31137-RE(twin)
FBgn0051441	CG31441 gene product from transcript CG31441-RB(CG31441)
FBgn0025683	CG3164 gene product from transcript CG3164-RA(CG3164)
FBgn0051784	CG31784 gene product from transcript CG31784-RC(CG31784)
FBgn0052081	CG32081 gene product from transcript CG32081-RA(CG32081)
FBgn0052369	CG32369 gene product from transcript CG32369-RC(CG32369)
FBgn0052572	CG32572 gene product from transcript CG32572-RA(CG32572)
FBgn0064119	CG33934 gene product from transcript CG33934-RE(CG33934)
FBgn0085264	CG34235 gene product from transcript CG34235-RB(CG34235)
FBgn0085341	CG34312 gene product from transcript CG34312-RA(CG34312)
FBgn0085344	CG34315 gene product from transcript CG34315-RB(CG34315)
FBgn0085436	CG34407 gene product from transcript CG34407-RG(Not1)
FBgn0031881	CG3476 gene product from transcript CG3476-RA(CG3476)
FBgn0032981	CG3635 gene product from transcript CG3635-RB(CG3635)
FBgn0028892	CG4161 gene product from transcript CG4161-RB(CG4161)
FBgn0259739	CG42393 gene product from transcript CG42393-RA(CG42393)
FBgn0034598	CG4266 gene product from transcript CG4266-RD(CG4266)
FBgn0261570	CG42684 gene product from transcript CG42684-RB(CG42684)
FBgn0261859	CG42788 gene product from transcript CG42788-RC(CG42788)
FBgn0262009	CG42827 gene product from transcript CG42827-RC(CG42827)
FBgn0263024	CG43319 gene product from transcript CG43319-RA(CG43319)
FBgn0263048	CG43343 gene product from transcript CG43343-RB(CG43343)
FBgn0264307	CG43782 gene product from transcript CG43782-RH(orb2)
FBgn0039071	CG4434 gene product from transcript CG4434-RA(CG4434)
FBgn0266436	CG45066 gene product from transcript CG45066-RA(CG45066)
FBgn0038745	CG4538 gene product from transcript CG4538-RC(CG4538)
FBgn0267363	CG45760 gene product from transcript CG45760-RB(JYalpha)
FBgn0259795	CG4750 gene product from transcript CG4750-RC(loopin-1)
FBgn0037994	CG4810 gene product from transcript CG4810-RA(CG4810)
FBgn0270925	CG4836 gene product from transcript CG4836-RC(CG4836)
FBgn0034258	CG4954 gene product from transcript CG4954-RB(eIF3-S8)
FBgn0032471	CG5122 gene product from transcript CG5122-RA(CG5122)
FBgn0039425	CG5432 gene product from transcript CG5432-RA(CG5432)
FBgn0040508	CG5983 gene product from transcript CG5983-RB(ACXC)
FBgn0036180	CG6091 gene product from transcript CG6091-RF(CG6091)
FBgn0027582	CG6230 gene product from transcript CG6230-RA(CG6230)
FBgn0032632	CG6380 gene product from transcript CG6380-RB(CG6380)
FBgn0027615	CG6404 gene product from transcript CG6404-RA(CG6404)
FBgn0039451	CG6420 gene product from transcript CG6420-RB(CG6420)
FBgn0036702	CG6512 gene product from transcript CG6512-RB(CG6512)
FBgn0036403	CG6661 gene product from transcript CG6661-RA(CG6661)
FBgn0038957	CG7059 gene product from transcript CG7059-RA(CG7059)

FBgn0032650	CG7094 gene product from transcript CG7094-RA(CG7094)
FBgn0031728	CG7235 gene product from transcript CG7235-RA(Hsp60C)
FBgn0032314	CG7309 gene product from transcript CG7309-RC(CG7309)
FBgn0037188	CG7369 gene product from transcript CG7369-RA(CG7369)
FBgn0036765	CG7408 gene product from transcript CG7408-RB(CG7408)
FBgn0037135	CG7414 gene product from transcript CG7414-RC(CG7414)
FBgn0036415	CG7768 gene product from transcript CG7768-RA(CG7768)
FBgn0027554	CG8042 gene product from transcript CG8042-RA(CG8042)
FBgn0034013	CG8166 gene product from transcript CG8166-RB(unc-5)
FBgn0011660	CG8169 gene product from transcript CG8169-RA(Pms2)
FBgn0029175	CG8433 gene product from transcript CG8433-RB(Ext2)
FBgn0033734	CG8520 gene product from transcript CG8520-RA(CG8520)
FBgn0036398	CG9007 gene product from transcript CG9007-RB(upSET)
FBgn0030669	CG9240 gene product from transcript CG9240-RA(CG9240)
FBgn0028513	CG9254 gene product from transcript CG9254-RA(CG9254)
FBgn0032878	CG9316 gene product from transcript CG9316-RA(CG9316)
FBgn0032076	CG9510 gene product from transcript CG9510-RD(CG9510)
FBgn0031085	CG9570 gene product from transcript CG9570-RA(CG9570)
FBgn0038360	CG9590 gene product from transcript CG9590-RA(CG9590)
FBgn0029939	CG9650 gene product from transcript CG9650-RG(CG9650)
FBgn0038200	CG9920 gene product from transcript CG9920-RA(CG9920)
FBgn0263979	Chromatin assembly factor 1, p55 subunit(Caf1-55)
FBgn0000299	Collagen type IV(Cg25C)
FBgn0015509	Cullin 1(Cul1)
FBgn0261268	Cullin 3(Cul3)
FBgn0015623	Cytochrome P450 reductase(Cpr)
FBgn0038925	Cytochrome c heme lyase(Cchl)
FBgn0032833	Cytochrome c oxidase subunit 4(COX4)
FBgn0027835	Dodeca-satellite-binding protein 1(Dp1)
FBgn0035538	Dopamine/Ecdysteroid receptor(DopEcR)
FBgn0052274	Drosomycin-like 1(Drsl1)
FBgn0032292	ER Membrane protein Complex 3(EMC3)
FBgn0011589	Eag-like K[+] channel(Elk)
FBgn0000568	Ecdysone-induced protein 75B(Eip75B)
FBgn0000579	Enolase(Eno)
FBgn0000723	FER ortholog (H. sapiens)(FER)
FBgn0262743	Female sterile (2) Ketel(Fs(2)Ket)
FBgn0035498	Fermitin 1(Fit1)
FBgn0010225	Gelsolin(Gel)
FBgn0004913	Germ line transcription factor 1(Gnf1)
FBgn0005674	Glutamyl-prolyl-tRNA synthetase(Aats-glupro)
FBgn0011244	Heat shock protein 60 related(Hsp60B)
FBgn0015245	Heat shock protein 60(Hsp60)
FBgn0001217	Heat shock protein cognate 2(Hsc70-2)
FBgn0001215	Heterogeneous nuclear ribonucleoprotein at 98DE(Hrb98DE)
FBgn0013981	Histone H4 replacement(His4r)
FBgn0035142	Homeodomain interacting protein kinase(Hipk)

FBgn0260466	I'm not dead yet 2(Indy-2)
FBgn0026415	Imaginal disc growth factor 4(Idgf4)
FBgn0261624	Inhibitor-3(I-3)
FBgn0020412	JIL-1 kinase(JIL-1)
FBgn0267330	KDEL receptor(KdelR)
FBgn0004379	Kinesin-like protein at 67A(Klp67A)
FBgn0261618	La related protein(larp)
FBgn0086915	Male-specific transcript 77F(Mst77F)
FBgn0029154	Malic enzyme like-1(Menl-1)
FBgn0029153	Malic enzyme like-2(Menl-2)
FBgn0039140	Mitochondrial Rho(Miro)
FBgn0053208	Molecule interacting with CasL(Mical)
FBgn0261836	Muscle-specific protein 300 kDa(Msp300)
FBgn0033224	Nop17 like(Nop17l)
FBgn0039735	Nucleophosmin(Nph)
FBgn0038063	Octopamine beta2 receptor(Octbeta2R)
FBgn0036080	Odorant receptor 67d(Or67d)
FBgn0039684	Odorant-binding protein 99d(Obp99d)
FBgn0029891	PTEN-induced putative kinase 1(Pink1)
FBgn0033377	Phosphomannomutase 45A(Pmm45A)
FBgn0004901	Phosphoribosylamidotransferase(Prat)
FBgn0051025	Protein phosphatase 1c interacting protein 1(Ppi1)
FBgn0016693	Putative Achaete Scute Target 1(Past1)
FBgn0029745	Regulatory particle non-ATPase 13-related(Rpn13R)
FBgn0028688	Regulatory particle non-ATPase 7(Rpn7)
FBgn0028687	Regulatory particle triple-A ATPase 1(Rpt1)
FBgn0087002	Retinoid- and fatty acid-binding glycoprotein(Rfabg)
FBgn0264707	Rho guanine nucleotide exchange factor 3(RhoGEF3)
FBgn0037879	SCP-containing protein C(scpr-C)
FBgn0025702	Serine-arginine protein kinase at 79D(Srpk79D)
FBgn0051163	Shal K[+] channel interacting protein(SKIP)
FBgn0040623	Spase 12-subunit(Spase12)
FBgn0045770	Sperm-Leucylaminopeptidase 3(S-Lap3)
FBgn0052064	Sperm-Leucylaminopeptidase 4(S-Lap4)
FBgn0033860	Sperm-Leucylaminopeptidase 5(S-Lap5)
FBgn0033868	Sperm-Leucylaminopeptidase 7(S-Lap7)
FBgn0034132	Sperm-Leucylaminopeptidase 8(S-Lap8)
FBgn0033266	Suppressor of Cytokine Signaling at 44A(Socs44A)
FBgn0031623	TBP-associated factor 30kD subunit alpha-2(Taf12L)
FBgn0039668	TRC8 ortholog(Trc8)
FBgn0041182	Thioester-containing protein 2(Tep2)
FBgn0020371	Translocase inner membrane 17(Tim17b2)
FBgn0033357	Translocase of outer membrane 7(Tom7)
FBgn0032397	Translocase of outer membrane 70(Tom70)
FBgn0023143	Ubiquitin activating enzyme 1(Uba1)
FBgn0038862	Ubiquitin specific protease 8(Usp8)
FBgn0039226	Uracil-DNA degrading factor(Ude)

FBgn0004045	Yolk protein 1(Yp1)
FBgn0260486	Zizimin ortholog (H. sapiens)(Ziz)
FBgn0015574	alpha-Esterase-6(alpha-Est6)
FBgn0050476	aveugle(ave)
FBgn0040237	belphegor(bor)
FBgn0003889	beta-Tubulin at 85D(betaTub85D)
FBgn0023097	bonus(bon)
FBgn0000206	bride of sevenless(boss)
FBgn0260794	circadian trip(ctrip)
FBgn0011202	diaphanous(dia)
FBgn0029088	dispatched(disp)
FBgn0037463	don juan like(djl)
FBgn0000547	echinoid(ed)
FBgn0260634	eukaryotic translation initiation factor 4G2(eIF4G2)
FBgn0264560	gartenzwerg(garz)
FBgn0051992	gawky(gw)
FBgn0024234	glass bottom boat(gbb)
FBgn0011224	hephaestus(heph)
FBgn0002431	hyperplastic discs(hyd)
FBgn0261955	knockdown(kdn)
FBgn0005654	latheo(lat)
FBgn0001313	male fertility factor kl2(kl-2)
FBgn0260745	midline fasciclin(mfas)
FBgn0086442	mind bomb 2(mib2)
FBgn0027579	minotaur(mino)
FBgn0002873	mushroom body defect(mud)
FBgn0002917	narrow abdomen(na)
FBgn0004227	no on or off transient A(nonA)
FBgn0035461	nutcracker(ntc)
FBgn0041102	ocnus(ocn)
FBgn0038168	oocyte maintenance defects(omd)
FBgn0263102	pipsqueak(psq)
FBgn0004400	rhino(rhi)
FBgn0031878	septin interacting protein 2(sip2)
FBgn0013733	short stop(shot)
FBgn0263873	sickie(sick)
FBgn0037810	slender lobes(sle)
FBgn0029157	slingshot(ssh)
FBgn0086704	slow termination of phototransduction(stops)
FBgn0003464	small optic lobes(sol)
FBgn0003435	smooth(sm)
FBgn0024189	sticks and stones(sns)
FBgn0085447	still life(sif)
FBgn0015600	toucan(toc)

Species
Drosophila melanogaster
Drosophila melanogaster
Drosophila melanogaster
Drosophila melanogaster
Drosophila melanogaster
Drosophila melanogaster
Drosophila melanogaster
Drosophila melanogaster
Drosophila melanogaster
Drosophila melanogaster
Drosophila melanogaster
Drosophila melanogaster
Drosophila melanogaster
Drosophila melanogaster
Drosophila melanogaster
Drosophila melanogaster
Drosophila melanogaster
Drosophila melanogaster
Drosophila melanogaster
Drosophila melanogaster
Drosophila melanogaster
Drosophila melanogaster
Drosophila melanogaster
Drosophila melanogaster
Drosophila melanogaster
Drosophila melanogaster
Drosophila melanogaster
Drosophila melanogaster
Drosophila melanogaster
Drosophila melanogaster
Drosophila melanogaster
Drosophila melanogaster
Drosophila melanogaster
Drosophila melanogaster
Drosophila melanogaster
Drosophila melanogaster
Drosophila melanogaster
Drosophila melanogaster
Drosophila melanogaster
Drosophila melanogaster
Drosophila melanogaster
Drosophila melanogaster
Drosophila melanogaster

Drosophila melanogaster
Drosophila melanogaster
Drosophila melanogaster
Drosophila melanogaster
Drosophila melanogaster
Drosophila melanogaster
Drosophila melanogaster
Drosophila melanogaster
Drosophila melanogaster
Drosophila melanogaster
Drosophila melanogaster
Drosophila melanogaster
Drosophila melanogaster
Drosophila melanogaster
Drosophila melanogaster
Drosophila melanogaster
Drosophila melanogaster
Drosophila melanogaster
Drosophila melanogaster
Drosophila melanogaster
Drosophila melanogaster
Drosophila melanogaster
Drosophila melanogaster
Drosophila melanogaster
Drosophila melanogaster
Drosophila melanogaster
Drosophila melanogaster
Drosophila melanogaster
Drosophila melanogaster
Drosophila melanogaster
Drosophila melanogaster
Drosophila melanogaster
Drosophila melanogaster
Drosophila melanogaster
Drosophila melanogaster
Drosophila melanogaster
Drosophila melanogaster
Drosophila melanogaster
Drosophila melanogaster
Drosophila melanogaster
Drosophila melanogaster
Drosophila melanogaster
Drosophila melanogaster
Drosophila melanogaster
Drosophila melanogaster
Drosophila melanogaster
Drosophila melanogaster

Drosophila melanogaster
Drosophila melanogaster
Drosophila melanogaster
Drosophila melanogaster
Drosophila melanogaster
Drosophila melanogaster
Drosophila melanogaster
Drosophila melanogaster
Drosophila melanogaster
Drosophila melanogaster
Drosophila melanogaster
Drosophila melanogaster
Drosophila melanogaster
Drosophila melanogaster
Drosophila melanogaster
Drosophila melanogaster
Drosophila melanogaster
Drosophila melanogaster
Drosophila melanogaster
Drosophila melanogaster
Drosophila melanogaster
Drosophila melanogaster
Drosophila melanogaster
Drosophila melanogaster
Drosophila melanogaster
Drosophila melanogaster
Drosophila melanogaster
Drosophila melanogaster
Drosophila melanogaster
Drosophila melanogaster
Drosophila melanogaster
Drosophila melanogaster
Drosophila melanogaster
Drosophila melanogaster
Drosophila melanogaster
Drosophila melanogaster
Drosophila melanogaster
Drosophila melanogaster
Drosophila melanogaster
Drosophila melanogaster
Drosophila melanogaster
Drosophila melanogaster
Drosophila melanogaster
Drosophila melanogaster
Drosophila melanogaster
Drosophila melanogaster
Drosophila melanogaster

Drosophila melanogaster
Drosophila melanogaster
Drosophila melanogaster
Drosophila melanogaster
Drosophila melanogaster
Drosophila melanogaster
Drosophila melanogaster
Drosophila melanogaster
Drosophila melanogaster
Drosophila melanogaster
Drosophila melanogaster
Drosophila melanogaster
Drosophila melanogaster
Drosophila melanogaster
Drosophila melanogaster
Drosophila melanogaster
Drosophila melanogaster
Drosophila melanogaster
Drosophila melanogaster
Drosophila melanogaster
Drosophila melanogaster
Drosophila melanogaster
Drosophila melanogaster
Drosophila melanogaster
Drosophila melanogaster
Drosophila melanogaster
Drosophila melanogaster
Drosophila melanogaster
Drosophila melanogaster
Drosophila melanogaster
Drosophila melanogaster
Drosophila melanogaster
Drosophila melanogaster
Drosophila melanogaster
Drosophila melanogaster
Drosophila melanogaster
Drosophila melanogaster
Drosophila melanogaster
Drosophila melanogaster
Drosophila melanogaster
Drosophila melanogaster
Drosophila melanogaster
Drosophila melanogaster
Drosophila melanogaster
Drosophila melanogaster
Drosophila melanogaster
Drosophila melanogaster

Drosophila melanogaster
Drosophila melanogaster
Drosophila melanogaster
Drosophila melanogaster
Drosophila melanogaster
Drosophila melanogaster
Drosophila melanogaster
Drosophila melanogaster
Drosophila melanogaster
Drosophila melanogaster
Drosophila melanogaster
Drosophila melanogaster
Drosophila melanogaster
Drosophila melanogaster
Drosophila melanogaster
Drosophila melanogaster
Drosophila melanogaster
Drosophila melanogaster
Drosophila melanogaster
Drosophila melanogaster
Drosophila melanogaster
Drosophila melanogaster
Drosophila melanogaster
Drosophila melanogaster
Drosophila melanogaster
Drosophila melanogaster
Drosophila melanogaster
Drosophila melanogaster
Drosophila melanogaster
Drosophila melanogaster
Drosophila melanogaster
Drosophila melanogaster
Drosophila melanogaster
Drosophila melanogaster
Drosophila melanogaster
Drosophila melanogaster
Drosophila melanogaster
Drosophila melanogaster
Drosophila melanogaster
Drosophila melanogaster
Drosophila melanogaster
Drosophila melanogaster
Drosophila melanogaster
Drosophila melanogaster

GOTERM_BP_DIRECT
GO:0006471~protein ADP-ribosylation,GO:0007264~small GTPase mediated signal transduction,GO:00
GO:0006085~acetyl-CoA biosynthetic process,GO:0006101~citrate metabolic process,GO:0006629~lipi
GO:0007618~mating,GO:0018991~oviposition,GO:0030728~ovulation,GO:0032504~multicellular organi
GO:0016567~protein ubiquitination,GO:0030071~regulation of mitotic metaphase/anaphase transition,G
GO:0006915~apoptotic process,GO:0010623~programmed cell death involved in cell development,GO:(
GO:0006289~nucleotide-excision repair,GO:0043161~proteasome-mediated ubiquitin-dependent proteir
GO:0055114~oxidation-reduction process,
GO:0006468~protein phosphorylation,GO:0006897~endocytosis,GO:0008360~regulation of cell shape,C
GO:0006152~purine nucleoside catabolic process,
GO:0009314~response to radiation,GO:0030308~negative regulation of cell growth,GO:0043065~positiv
GO:0006508~proteolysis,
GO:0042048~olfactory behavior,GO:0042384~cilium assembly,GO:0060271~cilium morphogenesis,
GO:0006506~GPI anchor biosynthetic process,
GO:0006839~mitochondrial transport,GO:0007399~nervous system development,
GO:0022416~chaeta development,GO:0035220~wing disc development,
GO:0051292~nuclear pore complex assembly,
GO:0006508~proteolysis,
GO:0006626~protein targeting to mitochondrion,GO:0016031~tRNA import into mitochondrion,GO:0030
GO:0035071~salivary gland cell autophagic cell death,GO:0048102~autophagic cell death,
GO:0006139~nucleobase-containing compound metabolic process,
GO:0030488~tRNA methylation,
GO:0006508~proteolysis,
GO:0009058~biosynthetic process,
GO:0006629~lipid metabolic process,GO:0016042~lipid catabolic process,GO:0032504~multicellular org
GO:0006811~ion transport,GO:0006839~mitochondrial transport,GO:0019233~sensory perception of pa
GO:0055085~transmembrane transport,
GO:0055085~transmembrane transport,
GO:0006171~cAMP biosynthetic process,GO:0007189~adenylate cyclase-activating G-protein coupled r
GO:0007498~mesoderm development,
GO:0000278~mitotic cell cycle,GO:0007094~mitotic spindle assembly checkpoint,GO:0007346~regulatic
GO:0000462~maturation of SSU-rRNA from tricistronic rRNA transcript (SSU-rRNA, 5.8S rRNA, LSU-rR

GO:0046949~fatty-acyl-CoA biosynthetic process,
GO:0008033~tRNA processing,GO:0030488~tRNA methylation,
GO:0006412~translation,GO:0032790~ribosome disassembly,GO:0045089~positive regulation of innate
GO:0006479~protein methylation,GO:2001020~regulation of response to DNA damage stimulus,

GO:0000289~nuclear-transcribed mRNA poly(A) tail shortening,GO:0006402~mRNA catabolic process,

GO:0003333~amino acid transmembrane transport,
GO:0006508~proteolysis,
GO:0002121~inter-male aggressive behavior,GO:0007476~imaginal disc-derived wing morphogenesis,G
GO:0006814~sodium ion transport,GO:0055085~transmembrane transport,GO:0071422~succinate tran

GO:0000289~nuclear-transcribed mRNA poly(A) tail shortening,GO:0006402~mRNA catabolic process,
GO:0006412~translation,GO:0006839~mitochondrial transport,GO:0006844~acyl carnitine transport,GO
GO:0006629~lipid metabolic process,GO:0016042~lipid catabolic process,
GO:0006479~protein methylation,

GO:0002121~inter-male aggressive behavior,GO:0007165~signal transduction,GO:0007476~imaginal d

GO:0032504~multicellular organism reproduction,
GO:0005975~carbohydrate metabolic process,GO:0006072~glycerol-3-phosphate metabolic process,G
GO:0007141~male meiosis I,GO:0007283~spermatogenesis,GO:0007288~sperm axoneme assembly,G
GO:0006520~cellular amino acid metabolic process,GO:0007005~mitochondrion organization,GO:0007

GO:0006457~protein folding,GO:0006508~proteolysis,
GO:0006812~cation transport,GO:0006813~potassium ion transport,GO:0006814~sodium ion transport,
GO:0006508~proteolysis,
GO:0001731~formation of translation preinitiation complex,GO:0006413~translational initiation,GO:0006
GO:0055114~oxidation-reduction process,
GO:0001731~formation of translation preinitiation complex,GO:0006413~translational initiation,GO:0006

GO:0006096~glycolytic process,
GO:0006171~cAMP biosynthetic process,GO:0007189~adenylate cyclase-activating G-protein coupled r
GO:0006909~phagocytosis,GO:0016579~protein deubiquitination,
GO:0006812~cation transport,GO:0006874~cellular calcium ion homeostasis,
GO:0009966~regulation of signal transduction,GO:0043666~regulation of phosphoprotein phosphatase
GO:0008535~respiratory chain complex IV assembly,GO:0051205~protein insertion into membrane,
GO:0016579~protein deubiquitination,
GO:0006461~protein complex assembly,GO:0006508~proteolysis,GO:0008053~mitochondrial fusion,G
GO:0006560~proline metabolic process,GO:0055114~oxidation-reduction process,
GO:0006094~gluconeogenesis,GO:0006096~glycolytic process,GO:0019233~sensory perception of pai

GO:0006468~protein phosphorylation,GO:0006909~phagocytosis,GO:0008360~regulation of cell shape
GO:0006458~'de novo' protein folding,GO:0006626~protein targeting to mitochondrion,GO:0007283~sp
GO:0006814~sodium ion transport,GO:0055085~transmembrane transport,GO:0071422~succinate tran
GO:0007264~small GTPase mediated signal transduction,GO:0008360~regulation of cell shape,GO:004
GO:0008152~metabolic process,GO:0042742~defense response to bacterium,
GO:0006417~regulation of translation,
GO:0000413~protein peptidyl-prolyl isomerization,GO:0006457~protein folding,

GO:0007165~signal transduction,GO:0007411~axon guidance,GO:0007432~salivary gland boundary sp
GO:0006298~mismatch repair,
GO:0006024~glycosaminoglycan biosynthetic process,GO:0006044~N-acetylglucosamine metabolic pro
GO:0019233~sensory perception of pain,
GO:0006343~establishment of chromatin silencing,GO:0016573~histone acetylation,GO:0048477~ooge
GO:0006465~signal peptide processing,GO:0006627~protein processing involved in protein targeting to
GO:0006820~anion transport,GO:0055085~transmembrane transport,
GO:0016567~protein ubiquitination,GO:0031146~SCF-dependent proteasomal ubiquitin-dependent prot
GO:0006526~arginine biosynthetic process,GO:0042450~arginine biosynthetic process via ornithine,GO

GO:0006355~regulation of transcription, DNA-templated,GO:0006366~transcription from RNA polymera
GO:0006458~'de novo' protein folding,GO:0006986~response to unfolded protein,GO:0051085~chaperc
GO:0000122~negative regulation of transcription from RNA polymerase II promoter,GO:0000281~mitotic
GO:0007391~dorsal closure,GO:0035848~oviduct morphogenesis,GO:0048621~post-embryonic digesti
GO:0006511~ubiquitin-dependent protein catabolic process,GO:0007095~mitotic G2 DNA damage chec
GO:0001745~compound eye morphogenesis,GO:0006511~ubiquitin-dependent protein catabolic proces
GO:0055114~oxidation-reduction process,
GO:0018063~cytochrome c-heme linkage,
GO:0000278~mitotic cell cycle,GO:0006123~mitochondrial electron transport, cytochrome c to oxygen,G
GO:0007059~chromosome segregation,GO:0030261~chromosome condensation,GO:0031507~heteroc
GO:0007186~G-protein coupled receptor signaling pathway,GO:0007213~G-protein coupled acetylcholi
GO:0050832~defense response to fungus,
GO:0016063~rhodopsin biosynthetic process,GO:0034975~protein folding in endoplasmic reticulum,GO
GO:0000160~phosphorelay signal transduction system,GO:0006813~potassium ion transport,GO:0010
GO:0000122~negative regulation of transcription from RNA polymerase II promoter,GO:0006351~transc
GO:0006096~glycolytic process,GO:0010906~regulation of glucose metabolic process,
GO:0006468~protein phosphorylation,GO:0007169~transmembrane receptor protein tyrosine kinase sig
GO:0000059~protein import into nucleus, docking,GO:0000060~protein import into nucleus, translocatio
GO:0007160~cell-matrix adhesion,GO:0048738~cardiac muscle tissue development,GO:0050829~defe
GO:0006909~phagocytosis,GO:0030041~actin filament polymerization,GO:0045010~actin nucleation,G
GO:0006260~DNA replication,GO:0006281~DNA repair,GO:0006351~transcription, DNA-templated,GO
GO:0006424~glutamyl-tRNA aminoacylation,GO:0006433~prolyl-tRNA aminoacylation,
GO:0006458~'de novo' protein folding,GO:0006626~protein targeting to mitochondrion,GO:0007286~sp
GO:0006458~'de novo' protein folding,GO:0006626~protein targeting to mitochondrion,GO:0007005~mi
GO:0006457~protein folding,
GO:0000381~regulation of alternative mRNA splicing, via spliceosome,GO:0001745~compound eye mo
GO:0006333~chromatin assembly or disassembly,GO:0006334~nucleosome assembly,GO:0006352~D
GO:0006468~protein phosphorylation,GO:0008586~imaginal disc-derived wing vein morphogenesis,GO

GO:0006814~sodium ion transport,GO:0006848~pyruvate transport,GO:0008340~determination of adult
GO:0005975~carbohydrate metabolic process,GO:0006032~chitin catabolic process,GO:0007444~imag

GO:0000723~telomere maintenance,GO:0006325~chromatin organization,GO:0006351~transcription, D
GO:0006621~protein retention in ER lumen,GO:0006890~retrograde vesicle-mediated transport, Golgi t
GO:0000070~mitotic sister chromatid segregation,GO:0007018~microtubule-based movement,GO:0007
GO:0000001~mitochondrion inheritance,GO:0007053~spindle assembly involved in male meiosis,GO:00
GO:0007290~spermatid nucleus elongation,GO:0035092~sperm chromatin condensation,GO:0035093~
GO:0006108~malate metabolic process,GO:0055114~oxidation-reduction process,
GO:0006108~malate metabolic process,GO:0055114~oxidation-reduction process,
GO:0000226~microtubule cytoskeleton organization,GO:0007005~mitochondrion organization,GO:0007
GO:0007015~actin filament organization,GO:0007411~axon guidance,GO:0007526~larval somatic mus
GO:0006997~nucleus organization,GO:0007015~actin filament organization,GO:0007303~cytoplasmic t

GO:0006338~chromatin remodeling,GO:0035041~sperm chromatin decondensation,
GO:0006355~regulation of transcription, DNA-templated,GO:0007186~G-protein coupled receptor signa
GO:0007608~sensory perception of smell,GO:0007619~courtship behavior,GO:0019236~response to p
GO:0002121~inter-male aggressive behavior,GO:0007606~sensory perception of chemical stimulus,GO
GO:0000266~mitochondrial fission,GO:0000422~mitophagy,GO:0006468~protein phosphorylation,GO:0
GO:0005975~carbohydrate metabolic process,
GO:0006164~purine nucleotide biosynthetic process,GO:0006189~'de novo' IMP biosynthetic process,G

GO:0006897~endocytosis,GO:0007291~sperm individualization,GO:0007474~imaginal disc-derived win
GO:0006511~ubiquitin-dependent protein catabolic process,
GO:0022008~neurogenesis,GO:0043161~proteasome-mediated ubiquitin-dependent protein catabolic p
GO:0006974~cellular response to DNA damage stimulus,GO:0030433~ER-associated ubiquitin-depend
GO:0006810~transport,GO:0006869~lipid transport,GO:0007224~smoothened signaling pathway,GO:0
GO:0035011~melanotic encapsulation of foreign target,GO:0035023~regulation of Rho protein signal tra

GO:0006468~protein phosphorylation,GO:0007268~chemical synaptic transmission,GO:0042742~defen
GO:0007608~sensory perception of smell,
GO:0006465~signal peptide processing,GO:0045047~protein targeting to ER,
GO:0006508~proteolysis,
GO:0006508~proteolysis,
GO:0006508~proteolysis,GO:0007498~mesoderm development,
GO:0006508~proteolysis,GO:0032504~multicellular organism reproduction,
GO:0006508~proteolysis,
GO:0006469~negative regulation of protein kinase activity,GO:0019221~cytokine-mediated signaling pa
GO:0006367~transcription initiation from RNA polymerase II promoter,
GO:0000209~protein polyubiquitination,GO:0007418~ventral midline development,GO:0016567~protein
GO:0006909~phagocytosis,GO:0050829~defense response to Gram-negative bacterium,
GO:0006626~protein targeting to mitochondrion,GO:0030150~protein import into mitochondrial matrix,
GO:0006626~protein targeting to mitochondrion,GO:0030150~protein import into mitochondrial matrix,G
GO:0006626~protein targeting to mitochondrion,
GO:0000209~protein polyubiquitination,GO:0006914~autophagy,GO:0008283~cell proliferation,GO:000
GO:0006511~ubiquitin-dependent protein catabolic process,GO:0008587~imaginal disc-derived wing m
GO:0006308~DNA catabolic process,GO:0035074~pupation,

GO:0007548~sex differentiation,
GO:0007264~small GTPase mediated signal transduction,GO:0035023~regulation of Rho protein signal
GO:0007158~neuron cell-cell adhesion,GO:0007416~synapse assembly,GO:0050804~modulation of sy
GO:0001751~compound eye photoreceptor cell differentiation,GO:0001754~eye photoreceptor cell diffe

GO:0007017~microtubule-based process,GO:0007435~salivary gland morphogenesis,
GO:0006325~chromatin organization,GO:0007411~axon guidance,GO:0007422~peripheral nervous sys
GO:0007165~signal transduction,GO:0007186~G-protein coupled receptor signaling pathway,GO:00071
GO:0022008~neurogenesis,GO:0042753~positive regulation of circadian rhythm,GO:0042787~protein u
GO:0000281~mitotic cytokinesis,GO:0000915~actomyosin contractile ring assembly,GO:0003383~apica
GO:0006887~exocytosis,GO:0007224~smoothened signaling pathway,GO:0007275~multicellular organi

GO:0002009~morphogenesis of an epithelium,GO:0002121~inter-male aggressive behavior,GO:000715
GO:0000082~G1/S transition of mitotic cell cycle,GO:0007140~male meiosis,GO:0007283~spermatoger
GO:0000902~cell morphogenesis,GO:0002064~epithelial cell development,GO:0006909~phagocytosis,
GO:0000184~nuclear-transcribed mRNA catabolic process, nonsense-mediated decay,GO:0001700~em
GO:0007274~neuromuscular synaptic transmission,GO:0007391~dorsal closure,GO:0007419~ventral c
GO:0000398~mRNA splicing, via spliceosome,GO:0006397~mRNA processing,GO:0006974~cellular re
GO:0000209~protein polyubiquitination,GO:0007112~male meiosis cytokinesis,GO:0007283~spermatog
GO:0005975~carbohydrate metabolic process,GO:0006099~tricarboxylic acid cycle,GO:0006101~citrate
GO:0006260~DNA replication,GO:0006267~pre-replicative complex assembly involved in nuclear cell cy
GO:0003341~cilium movement,
GO:0007155~cell adhesion,GO:0007409~axonogenesis,GO:0030198~extracellular matrix organization,
GO:0000209~protein polyubiquitination,GO:0007520~myoblast fusion,GO:0046716~muscle cell cellular
GO:0007009~plasma membrane organization,GO:0008654~phospholipid biosynthetic process,GO:0034
GO:0000132~establishment of mitotic spindle orientation,GO:0007058~spindle assembly involved in fem
GO:0006811~ion transport,GO:0006816~calcium ion transport,GO:0008344~adult locomotory behavior,
GO:0000398~mRNA splicing, via spliceosome,GO:0006355~regulation of transcription, DNA-templated,
GO:0006919~activation of cysteine-type endopeptidase activity involved in apoptotic process,GO:00072
GO:0035971~peptidyl-histidine dephosphorylation,
GO:0022008~neurogenesis,GO:0034472~snRNA 3'-end processing,
GO:0006342~chromatin silencing,GO:0006357~regulation of transcription from RNA polymerase II prom
GO:0000122~negative regulation of transcription from RNA polymerase II promoter,GO:0030381~chorio

GO:0000226~microtubule cytoskeleton organization,GO:0001578~microtubule bundle formation,GO:000
GO:0007015~actin filament organization,GO:0007409~axonogenesis,GO:0007602~phototransduction,G
GO:0007000~nucleolus organization,GO:0007275~multicellular organism development,
GO:0000278~mitotic cell cycle,GO:0000902~cell morphogenesis,GO:0006470~protein dephosphorylatic
GO:0007605~sensory perception of sound,GO:0016059~deactivation of rhodopsin mediated signaling,G
GO:0006508~proteolysis,GO:0007399~nervous system development,GO:0007601~visual perception,GO
GO:0006397~mRNA processing,GO:0007411~axon guidance,GO:0008340~determination of adult lifesp
GO:0001745~compound eye morphogenesis,GO:0007155~cell adhesion,GO:0007156~homophilic cell a
GO:0030036~actin cytoskeleton organization,GO:0035023~regulation of Rho protein signal transduction
GO:0007476~imaginal disc-derived wing morphogenesis,GO:0030707~ovarian follicle cell development,

GOTERM_CC_DIRECT
GO:0005622~intracellular,GO:0005794~Golgi apparatus,GO:0098793~presynapse,
GO:0005737~cytoplasm,GO:0005811~lipid particle,GO:0005829~cytosol,GO:00093
GO:0005576~extracellular region,GO:0005615~extracellular space,
GO:0005680~anaphase-promoting complex,
GO:0005623~cell,GO:0005739~mitochondrion,GO:0005758~mitochondrial intermem
GO:0016272~prefoldin complex,
GO:0005634~nucleus,
GO:0005789~endoplasmic reticulum membrane,GO:0031090~organelle membrane
GO:0005634~nucleus,GO:0005737~cytoplasm,
GO:0005829~cytosol,
GO:0016021~integral component of membrane,
GO:0000151~ubiquitin ligase complex,GO:0005737~cytoplasm,GO:0005886~plasm
GO:0016021~integral component of membrane,
GO:0005929~cilium,GO:0072372~primary cilium,GO:0097542~ciliary tip,
GO:0016021~integral component of membrane,
GO:0005789~endoplasmic reticulum membrane,GO:0016021~integral component o
GO:0005739~mitochondrion,GO:0005856~cytoskeleton,
GO:0016021~integral component of membrane,
GO:0034399~nuclear periphery,
GO:0016021~integral component of membrane,
GO:0016021~integral component of membrane,
GO:0005742~mitochondrial outer membrane translocase complex,GO:0031307~inte
GO:0016021~integral component of membrane,
GO:0005737~cytoplasm,
GO:0005739~mitochondrion,GO:0005829~cytosol,
GO:0016021~integral component of membrane,
GO:0016021~integral component of membrane,
GO:0005615~extracellular space,
GO:0005739~mitochondrion,GO:0005741~mitochondrial outer membrane,
GO:0005741~mitochondrial outer membrane,
GO:0005741~mitochondrial outer membrane,
GO:0005622~intracellular,GO:0005886~plasma membrane,GO:0016021~integral co
GO:0000776~kinetochore,GO:0000922~spindle pole,GO:0005634~nucleus,GO:000
GO:0005730~nucleolus,GO:0032040~small-subunit processome,

GO:0017086~3-methyl-2-oxobutanoate dehydrogenase (lipoamide) complex,

GO:0005622~intracellular,

GO:0016021~integral component of membrane,
GO:0005737~cytoplasm,GO:0005829~cytosol,GO:0030014~CCR4-NOT complex,
GO:0005634~nucleus,
GO:0016020~membrane,GO:0016021~integral component of membrane,
GO:0005813~centrosome,GO:0016021~integral component of membrane,
GO:0016021~integral component of membrane,

;O:0016319~mushroom body development,
GO:0005887~integral component of plasma membrane,GO:0016020~membrane,G(
GO:0016021~integral component of membrane,
GO:0016021~integral component of membrane,
GO:0016021~integral component of membrane,
GO:0000932~cytoplasmic mRNA processing body,GO:0005737~cytoplasm,GO:003
GO:0005623~cell,GO:0005743~mitochondrial inner membrane,GO:0016021~integr

GO:0016021~integral component of membrane,

GO:0031235~intrinsic component of the cytoplasmic side of the plasma membrane,(
GO:0005856~cytoskeleton,

GO:0005615~extracellular space,
GO:0005829~cytosol,GO:0009331~glycerol-3-phosphate dehydrogenase complex,
GO:0005634~nucleus,GO:0005737~cytoplasm,GO:0005829~cytosol,GO:0030054~
GO:0005739~mitochondrion,GO:0005759~mitochondrial matrix,
GO:0016021~integral component of membrane,
GO:0005739~mitochondrion,
GO:0005886~plasma membrane,GO:0005887~integral component of plasma meml
GO:0005737~cytoplasm,
GO:0005852~eukaryotic translation initiation factor 3 complex,GO:0016282~eukary(

GO:0005739~mitochondrion,GO:0005829~cytosol,GO:0005852~eukaryotic translati

GO:0005622~intracellular,GO:0005886~plasma membrane,GO:0016021~integral c(

GO:0005887~integral component of plasma membrane,GO:0012505~endomembra
GO:0000164~protein phosphatase type 1 complex,
GO:0005740~mitochondrial envelope,GO:0016021~integral component of membran

GO:0005739~mitochondrion,GO:0005743~mitochondrial inner membrane,GO:0016(
GO:0005759~mitochondrial matrix,
GO:0005829~cytosol,

GO:0005634~nucleus,GO:0005737~cytoplasm,
GO:0005759~mitochondrial matrix,
GO:0005887~integral component of plasma membrane,GO:0016020~membrane,
GO:0005622~intracellular,

GO:0005737~cytoplasm,

GO:0012505~endomembrane system,
GO:0016021~integral component of membrane,
GO:0005875~microtubule associated complex,GO:0032300~mismatch repair compl
GO:0000139~Golgi membrane,GO:0005783~endoplasmic reticulum,GO:0005789~

GO:0005634~nucleus,GO:0005700~polytene chromosome,GO:0035327~transcripti
GO:0005743~mitochondrial inner membrane,GO:0016021~integral component of m
GO:0005886~plasma membrane,GO:0016021~integral component of membrane,
GO:0005737~cytoplasm,GO:0019005~SCF ubiquitin ligase complex,
GO:0005829~cytosol,
GO:0016021~integral component of membrane,
GO:0000137~Golgi cis cisterna,
GO:0005634~nucleus,GO:0005829~cytosol,
GO:0005759~mitochondrial matrix,
GO:0005634~nucleus,GO:0005667~transcription factor complex,GO:0005700~poly
GO:0005581~collagen trimer,GO:0005587~collagen type IV trimer,
GO:0005634~nucleus,GO:0005737~cytoplasm,GO:0019005~SCF ubiquitin ligase c
GO:0005737~cytoplasm,GO:0031463~Cul3-RING ubiquitin ligase complex,GO:003
GO:0005789~endoplasmic reticulum membrane,GO:0005811~lipid particle,GO:001
GO:0005739~mitochondrion,GO:0005743~mitochondrial inner membrane,
GO:0005739~mitochondrion,GO:0005751~mitochondrial respiratory chain complex
GO:0000792~heterochromatin,GO:0005634~nucleus,GO:0005701~polytene chrom
GO:0005887~integral component of plasma membrane,GO:0016021~integral comp
GO:0005576~extracellular region,
GO:0005783~endoplasmic reticulum,GO:0012505~endomembrane system,GO:001
GO:0005622~intracellular,GO:0005887~integral component of plasma membrane,
GO:0005634~nucleus,
GO:0000015~phosphopyruvate hydratase complex,GO:0005737~cytoplasm,GO:00
GO:0005886~plasma membrane,GO:0005912~adherens junction,GO:0019898~ext
GO:0005622~intracellular,GO:0005635~nuclear envelope,GO:0005643~nuclear po
GO:0005737~cytoplasm,
GO:0005576~extracellular region,GO:0005829~cytosol,GO:0005884~actin filament,
GO:0005634~nucleus,GO:0005663~DNA replication factor C complex,GO:0005875
GO:0005737~cytoplasm,GO:0017101~aminoacyl-tRNA synthetase multienzyme co
GO:0005759~mitochondrial matrix,
GO:0005737~cytoplasm,GO:0005739~mitochondrion,GO:0005743~mitochondrial i
GO:0005739~mitochondrion,
GO:0005634~nucleus,GO:0005703~polytene chromosome puff,GO:0019013~viral
GO:0000228~nuclear chromosome,GO:0000786~nucleosome,GO:0005634~nucleu
GO:0005634~nucleus,

GO:0005887~integral component of plasma membrane,
GO:0005576~extracellular region,

GO:0000228~nuclear chromosome,GO:0000790~nuclear chromatin,GO:0005634~n
GO:0005783~endoplasmic reticulum,GO:0005789~endoplasmic reticulum membran
GO:0000776~kinetochore,GO:0005634~nucleus,GO:0005737~cytoplasm,GO:0005
GO:0005737~cytoplasm,GO:0005739~mitochondrion,GO:0005829~cytosol,
GO:0000785~chromatin,GO:0005634~nucleus,GO:0005874~microtubule,GO:00361
GO:0016021~integral component of membrane,

GO:0031307~integral component of mitochondrial outer membrane,
GO:0005829~cytosol,GO:0005886~plasma membrane,GO:0043195~terminal bouto
GO:0005635~nuclear envelope,GO:0005640~nuclear outer membrane,GO:000573
GO:0005737~cytoplasm,GO:0005875~microtubule associated complex,
GO:0005634~nucleus,GO:0005737~cytoplasm,
GO:0005887~integral component of plasma membrane,GO:0016021~integral comp
GO:0005886~plasma membrane,GO:0005929~cilium,GO:0016021~integral compor
GO:0005576~extracellular region,
GO:0005737~cytoplasm,GO:0005739~mitochondrion,GO:0016006~Nebenkern,
GO:0005829~cytosol,
O:0006541~glutamine metabolic process,GO:0008340~determination of adult lifesp

GO:0071212~subsynaptic reticulum,
GO:0005634~nucleus,GO:0005737~cytoplasm,GO:0008541~proteasome regulator
GO:0000502~proteasome complex,GO:0005838~proteasome regulatory particle,GO
GO:0005700~polytene chromosome,GO:0005737~cytoplasm,GO:0005838~proteas
GO:0005576~extracellular region,GO:0005615~extracellular space,GO:0005811~lip
nsduction,
GO:0005576~extracellular region,
GO:0048786~presynaptic active zone,

GO:0005787~signal peptidase complex,GO:0016021~integral component of membr
GO:0005737~cytoplasm,
GO:0005737~cytoplasm,
GO:0005737~cytoplasm,
GO:0005615~extracellular space,GO:0005737~cytoplasm,
GO:0005737~cytoplasm,
GO:0005737~cytoplasm,
GO:0001673~male germ cell nucleus,GO:0005669~transcription factor TFIID compl
GO:0005783~endoplasmic reticulum,GO:0005789~endoplasmic reticulum membran
GO:0005615~extracellular space,
GO:0005744~mitochondrial inner membrane presequence translocase complex,GO
GO:0005742~mitochondrial outer membrane translocase complex,GO:0031307~int
GO:0005742~mitochondrial outer membrane translocase complex,
GO:0005634~nucleus,GO:0005829~cytosol,GO:0005886~plasma membrane,GO:0
GO:0005737~cytoplasm,

GO:0005576~extracellular region,GO:0005811~lipid particle,GO:0005875~microtub
GO:0005622~intracellular,
GO:0005887~integral component of plasma membrane,GO:0009986~cell surface,G
GO:0005737~cytoplasm,GO:0005886~plasma membrane,
GO:0005739~mitochondrion,
GO:0005737~cytoplasm,GO:0005874~microtubule,
GO:0000790~nuclear chromatin,GO:0005634~nucleus,
GO:0005771~multivesicular body,GO:0005887~integral component of plasma mem
GO:0005634~nucleus,GO:0005737~cytoplasm,
GO:0005737~cytoplasm,GO:0005856~cytoskeleton,GO:0005884~actin filament,GC
GO:0016020~membrane,GO:0016021~integral component of membrane,
GO:0005634~nucleus,GO:0016007~mitochondrial derivative,GO:0036126~sperm fla
GO:0005886~plasma membrane,GO:0005912~adherens junction,GO:0005913~cell
iesis,GO:0008315~meiotic G2/MI transition,GO:0035071~salivary gland cell autopha
GO:0000137~Golgi cis cisterna,GO:0005801~cis-Golgi network,GO:0005802~trans
GO:0000932~cytoplasmic mRNA processing body,
GO:0005576~extracellular region,GO:0005615~extracellular space,GO:0012505~er
GO:0005634~nucleus,GO:0005737~cytoplasm,GO:0005829~cytosol,GO:0030529~
GO:0005634~nucleus,GO:0005737~cytoplasm,
GO:0005739~mitochondrion,GO:0005759~mitochondrial matrix,GO:0005875~micro
GO:0000784~nuclear chromosome, telomeric region,GO:0005656~nuclear pre-repli
GO:0016021~integral component of membrane,GO:0030286~dynein complex,GO:0
GO:0005578~proteinaceous extracellular matrix,GO:0005615~extracellular space,G
GO:0005737~cytoplasm,GO:0030018~Z disc,GO:0031430~M band,
GO:0005739~mitochondrion,GO:0005741~mitochondrial outer membrane,GO:0005
GO:0005813~centrosome,GO:0005819~spindle,GO:0005875~microtubule associat
GO:0005886~plasma membrane,GO:0016020~membrane,GO:0016021~integral co
GO:0005634~nucleus,GO:0071011~precatalytic spliceosome,GO:0071013~catalytic
GO:0005737~cytoplasm,GO:0019005~SCF ubiquitin ligase complex,
GO:0005634~nucleus,GO:0005829~cytosol,
GO:0032039~integrator complex,
GO:0005634~nucleus,GO:0031519~PcG protein complex,
GO:0000785~chromatin,GO:0000792~heterochromatin,GO:0005634~nucleus,

GO:0000235~astral microtubule,GO:0005737~cytoplasm,GO:0005856~cytoskeleton
O:0045087~innate immune response,GO:0045494~photoreceptor cell maintenance,
GO:0005634~nucleus,GO:0005730~nucleolus,GO:0005819~spindle,GO:0005875~r
GO:0005737~cytoplasm,GO:0005856~cytoskeleton,GO:0045177~apical part of cell
GO:0005622~intracellular,
GO:0005622~intracellular,GO:0005737~cytoplasm,
GO:0005654~nucleoplasm,
GO:0005886~plasma membrane,GO:0005917~nephrocyte diaphragm,GO:0016021
GO:0005622~intracellular,GO:0030054~cell junction,GO:0045202~synapse,
GO:0000922~spindle pole,GO:0005634~nucleus,GO:0005813~centrosome,GO:000

GOTERM_MF_DIRECT
GO:0003956~NAD(P)+-protein-arginine ADP-ribosyltransferase activity,GO:0005525~GTP binding,
GO:0003878~ATP citrate synthase activity,GO:0005524~ATP binding,GO:0016829~lyase activity,GO:00
GO:0042802~identical protein binding,
GO:0004842~ubiquitin-protein transferase activity,
GO:0003677~DNA binding,GO:0016491~oxidoreductase activity,GO:0046983~protein dimerization activ
GO:0003684~damaged DNA binding,
GO:0004497~monooxygenase activity,GO:0005506~iron ion binding,GO:0016705~oxidoreductase activi
GO:0004672~protein kinase activity,GO:0004674~protein serine/threonine kinase activity,GO:0005524~
GO:0008477~purine nucleosidase activity,
GO:0031624~ubiquitin conjugating enzyme binding,GO:0032182~ubiquitin-like protein binding,GO:0097
GO:0004252~serine-type endopeptidase activity,GO:0016787~hydrolase activity,
GO:0016740~transferase activity,GO:0051377~mannose-ethanolamine phosphotransferase activity,
GO:0019894~kinesin binding,
GO:0016747~transferase activity, transferring acyl groups other than amino-acyl groups,
GO:0004222~metalloendopeptidase activity,
GO:0015450~P-P-bond-hydrolysis-driven protein transmembrane transporter activity,GO:0030943~mitoc
GO:0005509~calcium ion binding,
GO:0003676~nucleic acid binding,GO:0046872~metal ion binding,
GO:0004867~serine-type endopeptidase inhibitor activity,
GO:0005524~ATP binding,GO:0019205~nucleobase-containing compound kinase activity,
GO:0004252~serine-type endopeptidase activity,GO:0016787~hydrolase activity,
GO:0003824~catalytic activity,GO:0004021~L-alanine:2-oxoglutarate aminotransferase activity,GO:0030
GO:0004806~triglyceride lipase activity,GO:0016298~lipase activity,GO:0016788~hydrolase activity, acti
GO:0005261~cation channel activity,GO:0008308~voltage-gated anion channel activity,GO:0015288~po
GO:0015267~channel activity,
GO:0015267~channel activity,
GO:0004016~adenylate cyclase activity,GO:0016849~phosphorus-oxygen lyase activity,
5635~nuclear envelope,GO:0005643~nuclear pore,GO:0005654~nucleoplasm,GO:0005737~cytoplasm,
GO:0070181~small ribosomal subunit rRNA binding,

GO:0003863~3-methyl-2-oxobutanoate dehydrogenase (2-methylpropanoyl-transferring) activity,
GO:0008168~methyltransferase activity,GO:0008175~tRNA methyltransferase activity,
GO:0003924~GTPase activity,GO:0005525~GTP binding,
GO:0008757~S-adenosylmethionine-dependent methyltransferase activity,GO:0019899~enzyme binding

GO:0004535~poly(A)-specific ribonuclease activity,GO:0005515~protein binding,
GO:0003676~nucleic acid binding,GO:0008270~zinc ion binding,
GO:0005524~ATP binding,GO:0016887~ATPase activity,

GO:0015171~amino acid transmembrane transporter activity,
GO:0004176~ATP-dependent peptidase activity,GO:0008270~zinc ion binding,

GO:0005215~transporter activity,GO:0015137~citrate transmembrane transporter activity,GO:0015141~

GO:0004535~poly(A)-specific ribonuclease activity,GO:0005515~protein binding,GO:0032947~protein co
GO:0003735~structural constituent of ribosome,GO:0005476~carnitine:acyl carnitine antiporter activity,G
GO:0004806~triglyceride lipase activity,GO:0016788~hydrolase activity, acting on ester bonds,
GO:0008757~S-adenosylmethionine-dependent methyltransferase activity,GO:0051998~protein carboxy

GO:0000166~nucleotide binding,GO:0003676~nucleic acid binding,GO:0003729~mRNA binding,
GO:0005096~GTPase activator activity,

GO:0004867~serine-type endopeptidase inhibitor activity,

GO:0004367~glycerol-3-phosphate dehydrogenase [NAD+] activity,GO:0042803~protein homodimerizat
GO:0000166~nucleotide binding,GO:0000900~translation repressor activity, nucleic acid binding,GO:000
GO:0004352~glutamate dehydrogenase (NAD+) activity,GO:0004353~glutamate dehydrogenase [NAD(I

GO:0004252~serine-type endopeptidase activity,GO:0005524~ATP binding,GO:0016787~hydrolase acti
GO:0005391~sodium:potassium-exchanging ATPase activity,GO:0005524~ATP binding,GO:0046872~m
GO:0004177~aminopeptidase activity,GO:0008235~metalloexopeptidase activity,GO:0030145~mangane
GO:0003743~translation initiation factor activity,
GO:0003939~L-iditol 2-dehydrogenase activity,GO:0008270~zinc ion binding,GO:0016491~oxidoreducta
GO:0003743~translation initiation factor activity,GO:0031369~translation initiation factor binding,
GO:0004092~carnitine O-acetyltransferase activity,GO:0016746~transferase activity, transferring acyl gr
GO:0004332~fructose-bisphosphate aldolase activity,
GO:0004016~adenylate cyclase activity,GO:0016849~phosphorus-oxygen lyase activity,
GO:0004843~thiol-dependent ubiquitin-specific protease activity,
GO:0000166~nucleotide binding,GO:0005524~ATP binding,GO:0016887~ATPase activity,GO:0019829~
GO:0004864~protein phosphatase inhibitor activity,
ıe,

GO:0004176~ATP-dependent peptidase activity,GO:0004222~metalloendopeptidase activity,GO:000552
GO:0003842~1-pyrroline-5-carboxylate dehydrogenase activity,GO:0004029~aldehyde dehydrogenase (
GO:0004082~bisphosphoglycerate mutase activity,GO:0004083~bisphosphoglycerate 2-phosphatase ac

GO:0004672~protein kinase activity,GO:0004674~protein serine/threonine kinase activity,GO:0005524~/
GO:0005524~ATP binding,
GO:0005215~transporter activity,GO:0015137~citrate transmembrane transporter activity,GO:0015141~
GO:0005088~Ras guanyl-nucleotide exchange factor activity,
GO:0003943~N-acetylgalactosamine-4-sulfatase activity,GO:0008484~sulfuric ester hydrolase activity,
GO:0003743~translation initiation factor activity,
GO:0003755~peptidyl-prolyl cis-trans isomerase activity,

GO:0005043~netrin receptor activity involved in chemorepulsion,
GO:0003697~single-stranded DNA binding,GO:0005524~ATP binding,GO:0016887~ATPase activity,GC
GO:0005515~protein binding,GO:0008375~acetylglucosaminyltransferase activity,GO:0015020~glucuro
GO:0005524~ATP binding,GO:0016887~ATPase activity,
GO:0001047~core promoter binding,GO:0008168~methyltransferase activity,GO:0008270~zinc ion bindi
GO:0004252~serine-type endopeptidase activity,GO:0008236~serine-type peptidase activity,
GO:0005436~sodium:phosphate symporter activity,GO:0022857~transmembrane transporter activity,
GO:0004842~ubiquitin-protein transferase activity,
GO:0004056~argininosuccinate lyase activity,

GO:0017137~Rab GTPase binding,
GO:0003676~nucleic acid binding,GO:0003700~transcription factor activity, sequence-specific DNA bind
GO:0046872~metal ion binding,GO:0051082~unfolded protein binding,GO:0051087~chaperone binding,
GO:0005515~protein binding,GO:0018024~histone-lysine N-methyltransferase activity,GO:0031491~nuc
GO:0005201~extracellular matrix structural constituent,
GO:0031625~ubiquitin protein ligase binding,
GO:0004842~ubiquitin-protein transferase activity,GO:0031625~ubiquitin protein ligase binding,
GO:0003958~NADPH-hemoprotein reductase activity,GO:0010181~FMN binding,GO:0030586~[methion
GO:0004408~holocytochrome-c synthase activity,
GO:0004129~cytochrome-c oxidase activity,
GO:0003696~satellite DNA binding,GO:0003697~single-stranded DNA binding,GO:0003730~mRNA 3'-U
GO:0001588~dopamine neurotransmitter receptor activity, coupled via Gs,GO:0008227~G-protein coupl

6020~membrane,GO:0072546~ER membrane protein complex,
GO:0000155~phosphorelay sensor kinase activity,GO:0005249~voltage-gated potassium channel activit
GO:0003677~DNA binding,GO:0003700~transcription factor activity, sequence-specific DNA binding,GC
GO:0000287~magnesium ion binding,GO:0004634~phosphopyruvate hydratase activity,
GO:0004713~protein tyrosine kinase activity,GO:0004715~non-membrane spanning protein tyrosine kina
GO:0008139~nuclear localization sequence binding,GO:0008320~protein transmembrane transporter ac
GO:0050839~cell adhesion molecule binding,
GO:0003779~actin binding,GO:0005509~calcium ion binding,
GO:0003677~DNA binding,GO:0003689~DNA clamp loader activity,GO:0005524~ATP binding,
GO:0003723~RNA binding,GO:0004818~glutamate-tRNA ligase activity,GO:0004827~proline-tRNA ligas
GO:0005524~ATP binding,GO:0051082~unfolded protein binding,
GO:0005524~ATP binding,GO:0051082~unfolded protein binding,
GO:0005524~ATP binding,GO:0051082~unfolded protein binding,
GO:0000166~nucleotide binding,GO:0003676~nucleic acid binding,GO:0003729~mRNA binding,GO:004
GO:0003677~DNA binding,GO:0046982~protein heterodimerization activity,
GO:0003677~DNA binding,GO:0004672~protein kinase activity,GO:0004674~protein serine/threonine ki

GO:0015137~citrate transmembrane transporter activity,GO:0015141~succinate transmembrane transpo
GO:0004568~chitinase activity,GO:0008061~chitin binding,GO:0008084~imaginal disc growth factor rec
GO:0008157~protein phosphatase 1 binding,
GO:0004674~protein serine/threonine kinase activity,GO:0005524~ATP binding,GO:0035175~histone ki
GO:0005046~KDEL sequence binding,
GO:0003774~motor activity,GO:0003777~microtubule motor activity,GO:0005524~ATP binding,GO:000
GO:0003723~RNA binding,
126~sperm flagellum,
GO:0004471~malate dehydrogenase (decarboxylating) (NAD+) activity,GO:0046872~metal ion binding,G
GO:0004471~malate dehydrogenase (decarboxylating) (NAD+) activity,GO:0004473~malate dehydroger
GO:0000287~magnesium ion binding,GO:0003924~GTPase activity,GO:0005509~calcium ion binding,G
GO:0003779~actin binding,GO:0008270~zinc ion binding,GO:0016491~oxidoreductase activity,GO:0016
GO:0003779~actin binding,GO:0005515~protein binding,GO:0008092~cytoskeletal protein binding,GO:0
GO:0008157~protein phosphatase 1 binding,
GO:0042393~histone binding,
GO:0003700~transcription factor activity, sequence-specific DNA binding,GO:0004930~G-protein couple
GO:0004984~olfactory receptor activity,GO:0005549~odorant binding,GO:0016503~pheromone recepto
GO:0005549~odorant binding,
GO:0004672~protein kinase activity,GO:0004674~protein serine/threonine kinase activity,GO:0005515~p
GO:0000287~magnesium ion binding,GO:0004614~phosphoglucomutase activity,GO:0004615~phospho
GO:0004044~amidophosphoribosyltransferase activity,GO:0046872~metal ion binding,GO:0051536~iron
GO:0008157~protein phosphatase 1 binding,
GO:0005509~calcium ion binding,GO:0005525~GTP binding,
GO:0043130~ubiquitin binding,
GO:0005515~protein binding,
GO:0005524~ATP binding,GO:0016787~hydrolase activity,GO:0016887~ATPase activity,GO:0017025~
GO:0005102~receptor binding,GO:0005319~lipid transporter activity,GO:0005504~fatty acid binding,GO
GO:0005089~Rho guanyl-nucleotide exchange factor activity,

GO:0004672~protein kinase activity,GO:0005524~ATP binding,

GO:0008233~peptidase activity,
GO:0004177~aminopeptidase activity,GO:0008235~metalloexopeptidase activity,GO:0030145~mangane
GO:0004177~aminopeptidase activity,GO:0008235~metalloexopeptidase activity,GO:0030145~mangane
GO:0004177~aminopeptidase activity,GO:0008235~metalloexopeptidase activity,GO:0030145~mangane
GO:0004177~aminopeptidase activity,GO:0008235~metalloexopeptidase activity,GO:0030145~mangane
GO:0004177~aminopeptidase activity,GO:0008235~metalloexopeptidase activity,GO:0030145~mangane
GO:0004860~protein kinase inhibitor activity,
GO:0046982~protein heterodimerization activity,
GO:0004842~ubiquitin-protein transferase activity,GO:0005515~protein binding,GO:0008270~zinc ion bi
GO:0004866~endopeptidase inhibitor activity,
GO:0008566~mitochondrial protein-transporting ATPase activity,GO:0015450~P-P-bond-hydrolysis-drive
GO:0015450~P-P-bond-hydrolysis-driven protein transmembrane transporter activity,
GO:0015450~P-P-bond-hydrolysis-driven protein transmembrane transporter activity,
GO:0004839~ubiquitin activating enzyme activity,GO:0004842~ubiquitin-protein transferase activity,GO:0
GO:0004843~thiol-dependent ubiquitin-specific protease activity,
GO:0003677~DNA binding,GO:0003723~RNA binding,GO:0004844~uracil DNA N-glycosylase activity,G

GO:0052689~carboxylic ester hydrolase activity,
GO:0005089~Rho guanyl-nucleotide exchange factor activity,
GO:0004872~receptor activity,GO:0042043~neurexin family protein binding,GO:0052689~carboxylic est

GO:0005524~ATP binding,
GO:0003924~GTPase activity,GO:0005200~structural constituent of cytoskeleton,GO:0005525~GTP bir
GO:0003682~chromatin binding,GO:0004842~ubiquitin-protein transferase activity,GO:0008270~zinc ior
GO:0004888~transmembrane signaling receptor activity,GO:0004930~G-protein coupled receptor activit
GO:0004842~ubiquitin-protein transferase activity,GO:0008270~zinc ion binding,GO:0016874~ligase act
GO:0003779~actin binding,GO:0005515~protein binding,GO:0005546~phosphatidylinositol-4,5-bisphosp

agellum,
GO:0005515~protein binding,
GO:0000340~RNA 7-methylguanosine cap binding,GO:0003743~translation initiation factor activity,
GO:0005086~ARF guanyl-nucleotide exchange factor activity,
GO:0000166~nucleotide binding,GO:0003723~RNA binding,GO:0005515~protein binding,
GO:0005125~cytokine activity,GO:0005160~transforming growth factor beta receptor binding,GO:00080
GO:0000166~nucleotide binding,GO:0000900~translation repressor activity, nucleic acid binding,GO:000
GO:0003723~RNA binding,GO:0004842~ubiquitin-protein transferase activity,GO:0008270~zinc ion binc
GO:0004108~citrate (Si)-synthase activity,GO:0046912~transferase activity, transferring acyl groups, ac
GO:0003677~DNA binding,GO:0003688~DNA replication origin binding,
GO:0005524~ATP binding,GO:0008569~ATP-dependent microtubule motor activity, minus-end-directed
GO:0050839~cell adhesion molecule binding,
GO:0004842~ubiquitin-protein transferase activity,GO:0008270~zinc ion binding,GO:0032033~myosin II
GO:0004366~glycerol-3-phosphate O-acyltransferase activity,
GO:0003677~DNA binding,GO:0005515~protein binding,
GO:0005216~ion channel activity,GO:0005261~cation channel activity,
GO:0000166~nucleotide binding,GO:0000976~transcription regulatory region sequence-specific DNA bir
GO:0004842~ubiquitin-protein transferase activity,
GO:0008969~phosphohistidine phosphatase activity,

GO:0003677~DNA binding,GO:0031208~POZ domain binding,GO:0042803~protein homodimerization a
GO:0003682~chromatin binding,
GO:0005515~protein binding,
GO:0003779~actin binding,GO:0005509~calcium ion binding,GO:0005515~protein binding,GO:0008017
GO:0005524~ATP binding,
nicrotubule associated complex,
GO:0003677~DNA binding,GO:0003779~actin binding,GO:0004721~phosphoprotein phosphatase activi

GO:0004198~calcium-dependent cysteine-type endopeptidase activity,GO:0008270~zinc ion binding,
GO:0000166~nucleotide binding,GO:0003723~RNA binding,GO:0003729~mRNA binding,
GO:0005515~protein binding,
GO:0005057~receptor signaling protein activity,GO:0005089~Rho guanyl-nucleotide exchange factor ac
)5819~spindle,GO:0005938~cell cortex,GO:0030496~midbody,GO:0031965~nuclear membrane,

)3676~nucleic acid binding,GO:0003723~RNA binding,GO:0003729~mRNA binding,GO:0003730~mRN/

nosyltransferase activity,GO:0046872~metal ion binding,GO:0050508~glucuronosyl-N-acetylglucosaminy

cleosome binding,GO:0035035~histone acetyltransferase binding,GO:0042054~histone methyltransferas

):0003707~steroid hormone receptor activity,GO:0004879~RNA polymerase II transcription factor activity

ʒ709~oxidoreductase activity, acting on paired donors, with incorporation or reduction of molecular oxyge

ed receptor activity,GO:0004989~octopamine receptor activity,GO:0004993~G-protein coupled serotonin

A 3'-UTR binding,GO:0008135~translation factor activity, RNA binding,GO:0008494~translation

/l-proteoglycan 4-alpha-N-acetylglucosaminyltransferase activity,GO:0050509~N-acetylglucosar

e activity,GO:0042393~histone binding,GO:0042803~protein homodimerization activity,GO:004

', ligand-activated sequence-specific DNA binding,GO:0004887~thyroid hormone receptor activi

n, NAD(P)H as one donor, and incorporation of one atom of oxygen,GO:0043914~NADPH:sulfu

receptor activity,GO:0008227~G-protein coupled amine receptor activity,GO:0043565~sequenc

2826~histone deacetylase binding,GO:0046974~histone methyltransferase activity (H3-K9 spec

ty,GO:0008270~zinc ion binding,GO:0020037~heme binding,GO:0043565~sequence-specific D

cific),GO:0046976~histone methyltransferase activity (H3-K27 specific),GO:0070615~nucleosor

ne-dependent ATPase activity,
