# Supplementary material for: The Drosophila RNA Helicase Belle (DDX3) Non-Autonomously Suppresses Germline Tumorigenesis Via Regulation of a Specific mRNA Set
Source: Cells. 2020 Feb 26;9(3):550. doi: 10.3390/cells9030550 (PMC7140462; doi:10.3390/cells9030550)
Supplement: Supplementary file 1 [file cells-09-00550-s001.zip › cells-679442-proof supplementary/Table S3.pdf]

				this paper	
number	Gene ID	Gene	peak regions	CLIP1	CLIP2
1	CG9484	***hyd***	CDS	yes	yes
2	CG34407	***not1***	3'UTR, CDS	yes	yes
3	CG5378	***rpn7***	CDS	yes	yes
4	CG1782	***uba1***	CDS	yes	yes
5	CG4236	***caf1-55***	CDS	yes	yes
6	CG2637	***Fs(2)Ket***	3'UTR	yes	yes
7	CG8487	***garz***	CDS	yes	yes
8	CG6238	***ssh***	CDS	yes	yes
9	CG2331	***ter94***	5'UTR	yes	yes
10	CG11027	***arf102f***	CDS	yes	yes
11	CG1877	***cul1***	CDS	yes	yes
12	CG3186	***eIF5A***	CDS	yes	yes
13	CG42574	***ctrip***	CDS	yes	yes
14	CG1768	***dia***	CDS	yes	yes
15	CG7929	***ocn***	CDS	yes	yes
16	CG8114	***pbl***	CDS	yes	mc*
17	CG17603	***taf1***	CDS	yes	mc*
18	CG8416	***rho1***	3'UTR	yes	mc*
19	CG6699	***beta'COP***	CDS	yes	mc*
20	CG10110	***Cpsf160***	CDS	yes	mc*
21	CG2041	***lgs***	CDS	yes	mc*
22	CG9983	***hrb98de***	CDS	yes	yes

mc* medium confiden

Supplementary Table S3. List of genes, knockdowns of which provide the morphological defects in the testes according to data of three independent screens and which determined in our CLIP analysis as putative mRNA targets of Belle**.** RNAi knockdowns of 17 genes determined in CLIP analysis lead to similar phenotypes in case of *belKD* in somatic cyst cells or in germ cells according to the results of at least a one screen (matched by yellow and orange color). Four genes, *not1, rpn7, rho1,* and *caf1-55* , knockdowns of which provide the same morphological defects in the testes according to data of three independent screenings are marked by orange color.

screen tj-Gal4 x UAS-RNAi (testis)

**Fairchild et al. 2017(PMID:28957323)**
Cyst cells absent
Cyst cells absent
Cyst cells absent
Cyst cells absent
Cyst cells absent
Cyst cells absent
phenotype not shown, male sterility
spermatogonial defects
spermatid defects
spermatid defects
phenotype not shown, male sterility
no data
no data
no data
no data
Cyst cells absent
no data
Cyst cells absent
Cyst cells absent
no data
no data
no data

ce, see Material and Methods

screen tj-Gal4 x UAS-RNAi (testis)

Yu et al. 2016 (PMID:27471256)
Soma-induced germ cell differentiation defects
Soma-induced germ cell differentiation defects
Soma-induced germ cell differentiation defects
no data
Soma-induced germ cell maintenance defects
no data
no data
Soma-induced germ cell differentiation defects
no data
no data
no data
no data
no data
no data
Soma-induced germ cell maintenance defects
no data
Soma-induced germ cell differentiation defects
Soma-induced germ cell differentiation defects
Soma-induced germ cell differentiation defects
no data
no data
normal

screen nos-GAL4 x UAS-RNAi (testis)

**Yu et al. 2016 (PMID:27471256)**
GSC maintenance defects(no germ cell survived)
GSC maintenance defects(no germ cell survived)
GSC maintenance defects(no germ cell survived)
no data
GSC maintenance defects(a few germ cells survived)
no data
no data
GSC maintenance defects(a few germ cells survived)
no data
no data
no data
no data
no data
no data
GSC maintenance defects(no germ cell survived)
no data
GSC maintenance defects(a few germ cells survived)
GSC maintenance defects(no germ cell survived)
GSC maintenance defects(no germ cell survived)
no data
no data
GSC maintenance defects(a few germ cells survived)

screen Act5-Gal4, UAS-GFP/tub-Gal80ts/ x UAS-RNAi (testis)

**Liu et al. 2016 (PMID:27484291)**
low GSC increase
medium-low GSC decrease
medium-high GSC decrease
no data
high GSC decrease
no data
no data
no data
medium-high GSC decrease
no data
no data
medium-high GSC increase
medium-low GSC increase
medium-high GSC decrease
low GSC increase
high GSC decrease
medium-high GSC increase
high GSC decrease
no data
high GSC decrease
medium-high GSC decrease
no data

molecular functions
ubiquitination, protein stability / Hh
deadenylation complex
ubiquitination, protein stability, 26S proteasome
ubiquitin-activating enzyme E1
nucleosome remodelling and deacetylase (NuRD) complex, dREAM complex, tMAC complex
beta-importin, Ran GTPase binding
ARF guanyl-nucleotide exchange factor activity; Rab GTPase binding
actin cytoskeleton regulation, phosphatase, cofilin dephosphorilation
ubiquitin-proteasome system, ER formation, vesicle budding
member of Ras superfamily of small GTPases
E3 ubiquitin-protein ligase complexes
eukaryotic translation elongation factor 5
circadian trip/ubiquitin-protein ligase
Dia-class formin
protein histidine phosphatase activity, testis specific factor
Rho guanyl-nucleotide exchange factor
general transcription factor
small GTPase
Beta-coatomer protein of a cytosolic protein complex coatomer that binds to dilysine motifs
Cleavage and polyadenylation specificity factor 160
transcription coactivator
Heterogeneous nuclear ribonucleoprotein at 98DE, RNA-binding

pathway
Ubiquitin mediated proteolysis / Hh
RNA degradation, CCR4-Not complex
proteasome-mediated ubiquitin-dependent protein catabolic process
ubiquitination, protein stability
nucleosome remodeling factor, cell division, cytokinesis
nuclear import and export
endomembrane transport
protein tyrosine/serine/threonine phosphatase activity, actin binding
Wingless pathway, Heagehog pathway
ADP ribosylation factor 102F, endomembrane transport
negative regulation of TOR signaling, insulin signaling
translation elongation
ubiquitination, protein stability
cell division/cytokinesis
no data
actin cytoskeleton, cell adhesion
general transcription factor complex TFIID
actin cytoskeleton, cell adhesion
retrograde vesicle transport from the Golgi to ER.
Component of cleavage and polyadenylation specificity factor (CPSF) complex
signal transduction through the Wnt pathway
hnRNA stability, splicing, IRES-dependent translation, and translational repression

	CLIP analysis in HEK293T cells
**human homolog**	**Valentin-Vega et al.2016 (PMID:27180681)**
UBR5	yes, CDS
Cnot1	yes, CDS
PSMD6	no
UBA1(CFAP124, POC20, UBE1X)	no
RBBP4, RBBP7	yes, CDS
KPNB1	no
GBF1	no
SSH1, SSH2, SSH3	no
VCP	no
ARF4,ARF5	no
CUL1	yes, 5'UTR, CDS
EIF5A, EIF5A1, EIF5A2, EIF5AL1	yes, CDS
TRIP12	no
DIAPH3, DIAPH1, DIAPH2	no
PHPT1	no
ECT2	no
TAF1, TAF1L	no
RHOA, RHOC, RHOB	no
COPB1	yes, CDS
CPSF1	no
BCL9L	no
HNRNPA2B1	yes, CDS

Gene
hyd
not1
rpn7
uba1
caf1-55
Fs(2)Ket
garz
ssh
ter94
arf102f
cul1
eIF5A
ctrip
dia
ocn
pbl
taf1
rho1
beta'COP
Cpsf160
lgs
hrb98de
