# Supplementary material for: The Drosophila RNA Helicase Belle (DDX3) Non-Autonomously Suppresses Germline Tumorigenesis Via Regulation of a Specific mRNA Set
Source: Cells. 2020 Feb 26;9(3):550. doi: 10.3390/cells9030550 (PMC7140462; doi:10.3390/cells9030550)
Supplement: Supplementary file 1 [file cells-09-00550-s001.zip › cells-679442-proof supplementary/Table S4.pdf]

#clustering method	cluster number	cluster color	gene count
MCL	1	Red	4
MCL	1	Red	4
MCL	1	Red	4
MCL	1	Red	4
MCL	3	Green	4
MCL	3	Green	4
MCL	3	Green	4
MCL	3	Green	4
MCL	2	Yellow	4
MCL	2	Yellow	4
MCL	2	Yellow	4
MCL	2	Yellow	4
MCL	4	Cyan	3
MCL	4	Cyan	3
MCL	4	Cyan	3
MCL	5	Blue	2
MCL	5	Blue	2

protein name	protein identifier	protein description		
hyd	7227.FBpp0081568	E3 ubiquitin-protein ligase hyd; E3 ubiquitin-prot		
ctrip	7227.FBpp0292914	Circadian trip (Ctrip) is a HECT ubiquitin ligase t		
Uba1	7227.FBpp0087583	ATP binding; ubiquitin activating enzyme activity		
Taf1	7227.FBpp0293442	Transcription initiation factor TFIID subunit 1; TF		
Rpn7	7227.FBpp0083687	26S proteasome non-ATPase regulatory subunit		
TER94	7227.FBpp0111818	Transitional endoplasmic reticulum ATPase TER		
Cul1	7227.FBpp0087921	Cullin homolog 1; Core component of multiple S		
Fs(2)Ket	7227.FBpp0294035	Importin subunit beta; Required for nuclear prote		
garz	7227.FBpp0087010	Gartenzwerg, isoform B; Gartenzwerg (Garz) is a		
beta'COP	7227.FBpp0080048	Coatomer subunit beta; The coatomer is a cytos		
Not1	7227.FBpp0293496	Not1, isoform C; Protein binding. It is involved in		
Arf102F	7227.FBpp0088256	ADP-ribosylation factor 2; GTP-binding protein in		
Rho1	7227.FBpp0086354	Ras-like GTP-binding protein Rho1; Has a role in		
dia	7227.FBpp0297103	Protein diaphanous; Required for cytokinesis in		
pbl	7227.FBpp0076487	Pebble, isoform B; Semaphorin receptor binding		
Cpsf160	7227.FBpp0089163	Cleavage and polyadenylation specificity factor s		
Hrb98DE	7227.FBpp0084669	Heterogeneous nuclear ribonucleoprotein at 98D		

ein ligase which accepts ubiquitin from an E2 ubiquitin-conjugating enzyme in the form o
hat regulates the levels and circadian oscillations of the Clk transcriptional activator. Ctri
. It is involved in the biological process described with: cell proliferation; neuron remodel
FIID is a multimeric protein complex that plays a central role in mediating promoter respo
: 6; Acts as a regulatory subunit of the 26S proteasome which is involved in the ATP-dep
R94; Necessary for the fragmentation of Golgi stacks during mitosis and for their reassem
CF (SKP1-CUL1-F-box protein) E3 ubiquitin-protein ligase complexes which mediate the
ein import and mediates docking of import substrate to distinct nucleoporins. In Drosoph
a guanine nucleotide exchange factor localized to the cis-Golgi apparatus. Garz roles inc
olic protein complex that binds to dilysine motifs and reversibly associates with Golgi no
the biological process described with: mRNA catabolic process; muscle organ developm
nvolved in protein trafficking; may modulate vesicle budding and uncoating within the Go
n regulating actin cytoskeletal organization: required during early development for prope
both mitosis and meiosis. Has a role in actin cytoskeleton organization and is essential f
; GTPase activator activity; guanyl-nucleotide exchange factor activity; Rho GTPase bin
subunit 1; Component of the cleavage and polyadenylation specificity factor (CPSF) com
DE (Hrb98DE) is a nuclear RNA-binding protein. It controls hnRNA stability, splicing, IRE

of a thioester and then directly transfers the ubiquitin to targeted substrates (By similarity

ling; regulation of programmed cell death; regulation of Ras protein signal transduction;
onses to various activators and repressors. Largest component and core scaffold of the c

mbly after mitosis. Involved in the formation of the transitional endoplasmic reticulum (tE
e ubiquitination of proteins involved in cell cycle progression, signal transduction and tra
ila, may not function as a snRNP import receptor as it does not interact with components
clude membrane and protein trafficking between the Golgi apparatus and the endoplasm
n- clathrin-coated vesicles, which further mediate biosynthetic protein transport from the
ment; mitotic G2 DNA damage checkpoint; dendrite morphogenesis; neurogenesis; nucl

r execution of morphogenetic movements of individual cells and groups of cells importan
for many, if not all, actin-mediated events involving membrane invagination. May serve a
ding; Rac guanyl-nucleotide exchange factor activity; phosphatidylinositol phosphate bin
nplex that plays a key role in pre- mRNA 3'-end formation, recognizing the AAUAAA sign
ES-dependent translation, and translational repression. Hrb98DE represents one of the n

y). Required for regulation of cell proliferation in imaginal disks and germ cells. Acts as a
regulation of growth; autophagy; mushroom body development; larval midgut cell progra
complex. Contains N- and C-terminal Ser/Thr kinase domains which can autophosphory
R). The transfer of membranes from the endoplasmic reticulum to the Golgi apparatus c
nscription. In the SCF complex, serves as a rigid scaffold that organizes the SKP1-F-bo
e ER, via the Golgi up to the trans Golgi network. Coatomer complex is required for budc
ear-transcribed mRNA poly(A) tail shortening; negative regulation of translation
nt for the formation of the embryonic body plan. Plays a role in regulating dorsal closure
as a mediator between signaling molecules and actin organizers at specific phases of the
nal sequence and interacting with poly(A) polymerase and other factors to bring about cl
main targets of the poly(ADP-ribosyl)ation pathway. Hrb98DE also regulates tissue polar

a negative regulator of hh, ci and dpp expression in the anterior of the eye disk

ammed cell death; protein ubiquitination; lipid storage; Belongs to the ubiquitin-activating
ylate or transphosphorylate other transcription factors. Possesses DNA-binding activity. E

occurs via 50-70 nm transition vesicles which derive from part-rough, part-smooth transit
x protein and RBX1 subunits. May contribute to catalysis through positioning of the subs

ding from Golgi membranes, and is essential for the retrograde Golgi-to-ER transport of

during embryogenesis. During embryogenesis, acts upstream of wash to regulate the d
e cell cycle. Possible component of the contractile ring or may control its function

eavage and poly(A) addition. This subunit is involved in the RNA recognition step of the

Essential for progression of the G1 phase of the cell cycle. Negative regulator of the TAT
tional elements of the endoplasmic reticulum (tER). Vesicle budding from the tER is an A<br>strate and the ubiquitin-conjugating enzyme. During early metamorphosis, part of the SC
levelopmental migration of tail hemocytes anteriorly along the ventral midline. May have

ATP-dependent process. Involved in the ubiquitin-proteasome system. Important for osk
CF-slmb complex that negatively regulates the InR/PI3K/TOR pathway to activate the pru

kar mRNA localization and/ [...]
uning of unnecessary larva [...]
